# Supplementary material for: Integrated transcriptome and metabolome revealed the drought responsive metabolic pathways in Oriental Lily (Lilium L.)
Source: PeerJ. 2023 Dec 18;11:e16658. doi: 10.7717/peerj.16658 (PMC10734436; doi:10.7717/peerj.16658)
Supplement: Supplemental Information 1 [file peerj-11-16658-s001.docx]

Table 1 The effects of ck and drought on growth and leaf morphology of lily

| Time/day | Leaf lenght/cm | Leaf width/cm | lenght/widt ratio | Leaf area |
| --- | --- | --- | --- | --- |
| 10 | 0^a^ | 0^a^ | 0^a^ | 0^a^ |
|  | 0^a^ | 0^a^ | 0^a^ | 0^a^ |
| 15 | 6.2^a^ | 1.42^a^ | 4.36^a^ | 7.28^a^ |
|  | 4.8^b^ | 1.23^b^ | 3.91b | 4.36^b^ |
| 20 | 7.8^a^ | 1.51^a^ | 5.16^a^ | 11.51^a^ |
|  | 6.1^b^ | 1.24^b^ | 4.35^b^ | 7.03^b^ |
| 25 | 8.5^a^ | 1.52^a^ | 5.58^a^ | 13.66^a^ |
|  | 6.9^b^ | 1.24^b^ | 4.73^b^ | 8.49^b^ |

Note: Different lowercase letters indicate a significant difference (P <0.05).

Table 2 Statistics of in vitro leaf water loss rate of wild-type and transgenic plant lines.

| Time | 0 | 2 | 4 | 6 | 8 | 10 | 12 |
| --- | --- | --- | --- | --- | --- | --- | --- |
| ck | 0.00^b^ | 0.35^b^ | 0.70 ^b^ | 0.81^b^ | 0.85^b^ | 0.92^a^ | 0.95 ^a^ |
| TRINITY_DN2608-2 | 0.00^a^ | 0.44^a^ | 0.78^a^ | 0.87^a^ | 0.90^a^ | 0.94^a^ | 0.96^a^ |
| TRINITY_DN2608-3 | 0.00^a^ | 0.45^a^ | 0.79^a^ | 0.86^a^ | 0.89^a^ | 0.93^a^ | 0.95^a^ |
| TRINITY_DN2608-4 | 0.00^a^ | 0.43^a^ | 0.76^a^ | 0.87^a^ | 0.88^a^ | 0.93^a^ | 0.94^a^ |
