# Supplementary figures and images for: Integrated transcriptome and metabolome revealed the drought responsive metabolic pathways in Oriental Lily (Lilium L.)
Source: PeerJ. 2023 Dec 18;11:e16658. doi: 10.7717/peerj.16658 (PMC10734436; doi:10.7717/peerj.16658)

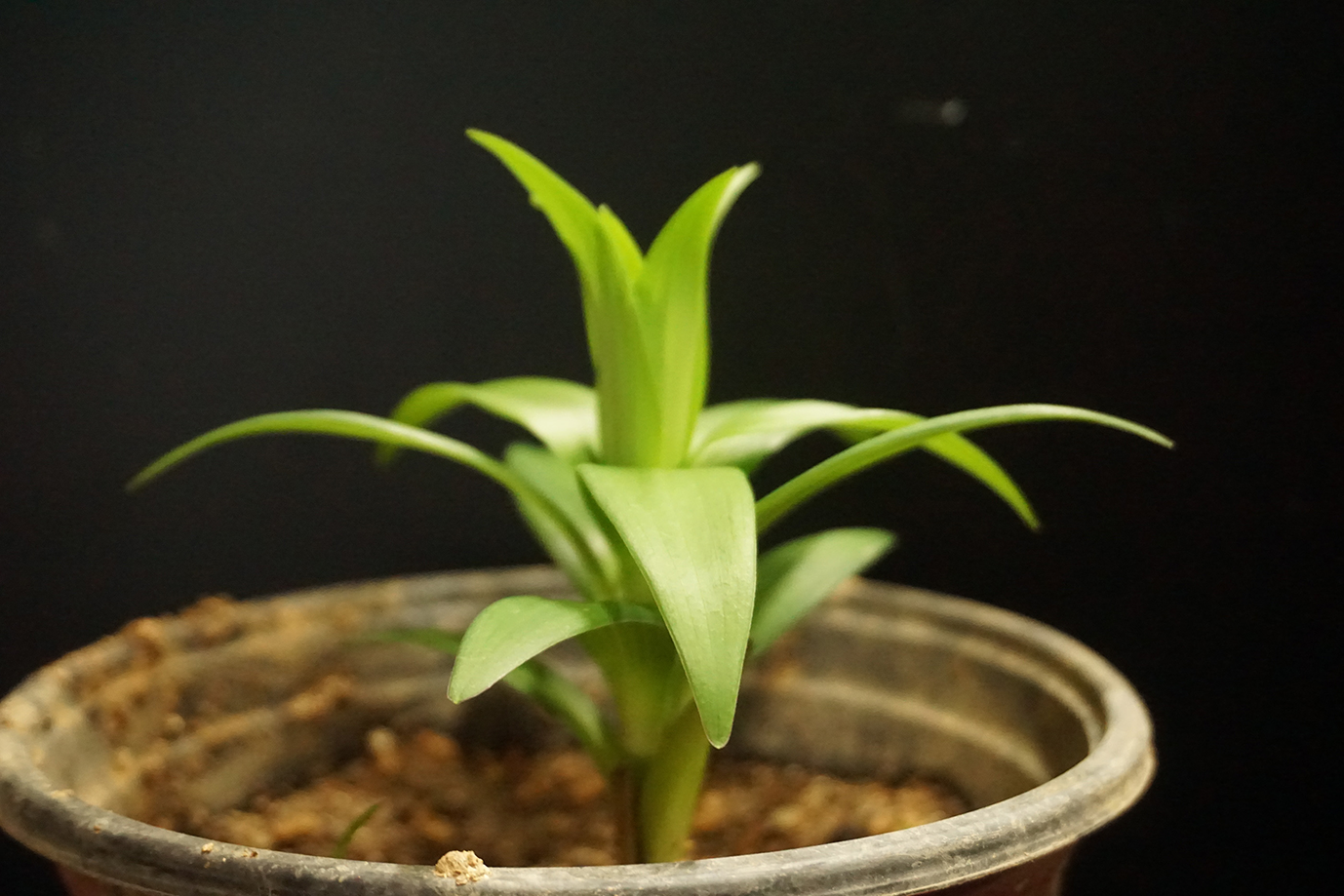

Supplement: Supplemental Information 2 [file peerj-11-16658-s002.zip › 7.27/Fig. 1/a.png]

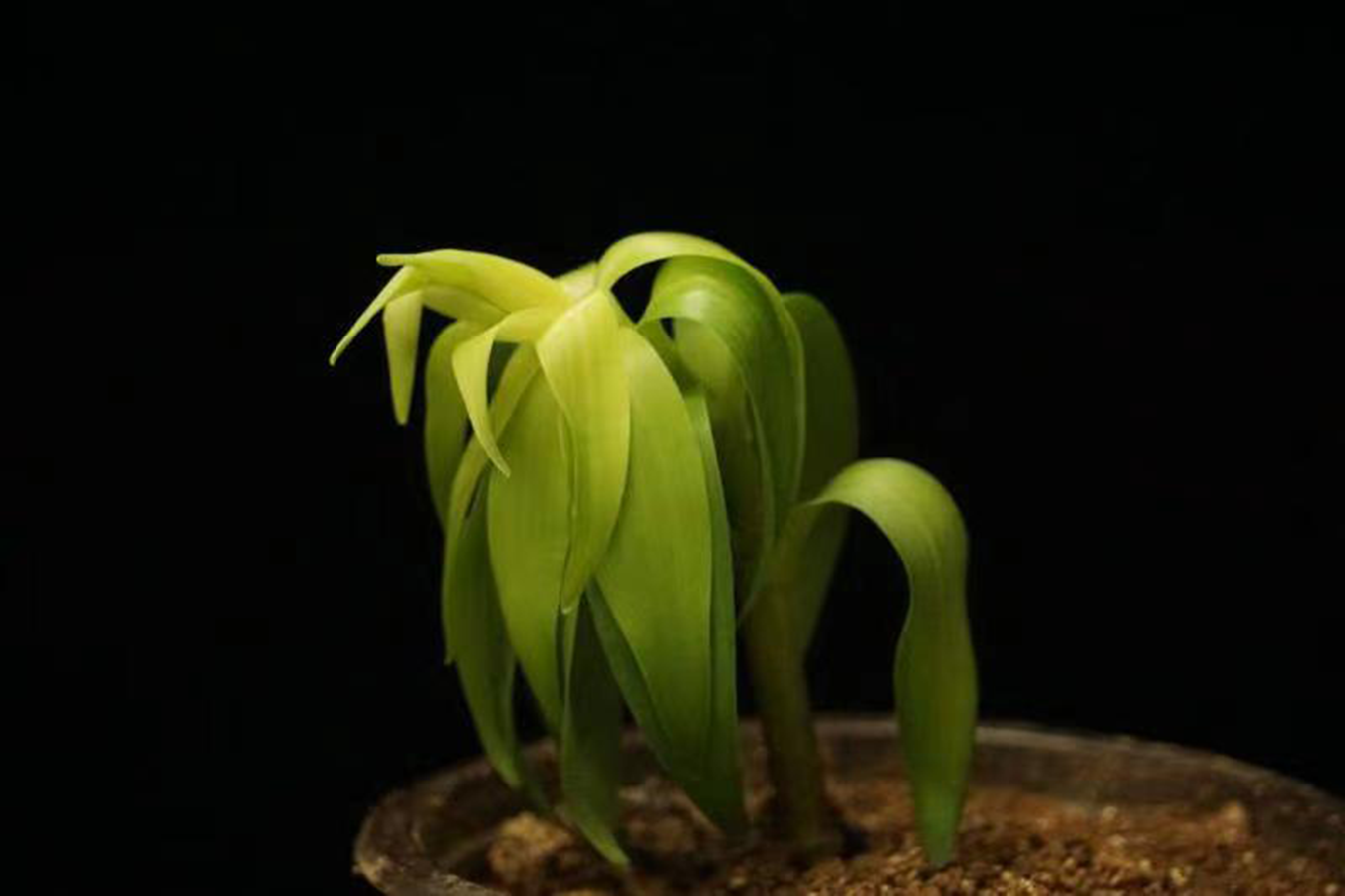

Supplement: Supplemental Information 2 [file peerj-11-16658-s002.zip › 7.27/Fig. 1/b.png]

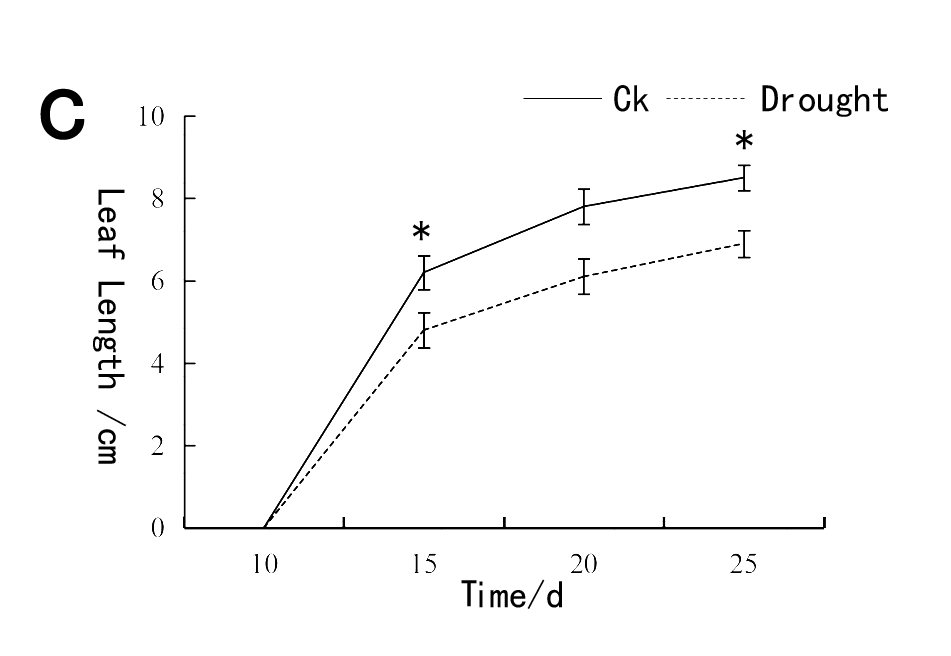

Supplement: Supplemental Information 2 [file peerj-11-16658-s002.zip › 7.27/Fig. 1/c.png]

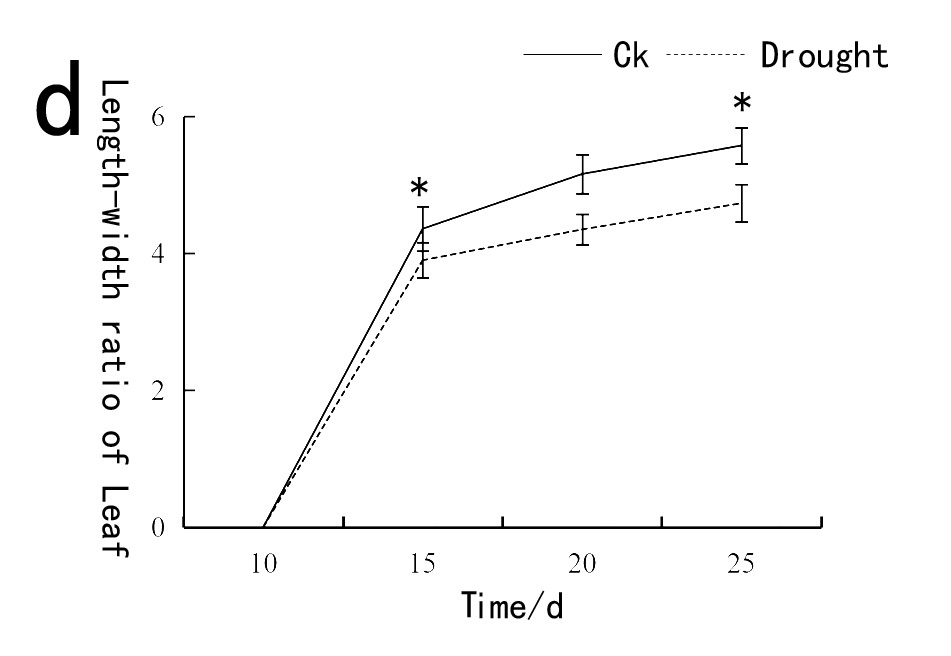

Supplement: Supplemental Information 2 [file peerj-11-16658-s002.zip › 7.27/Fig. 1/d.png]

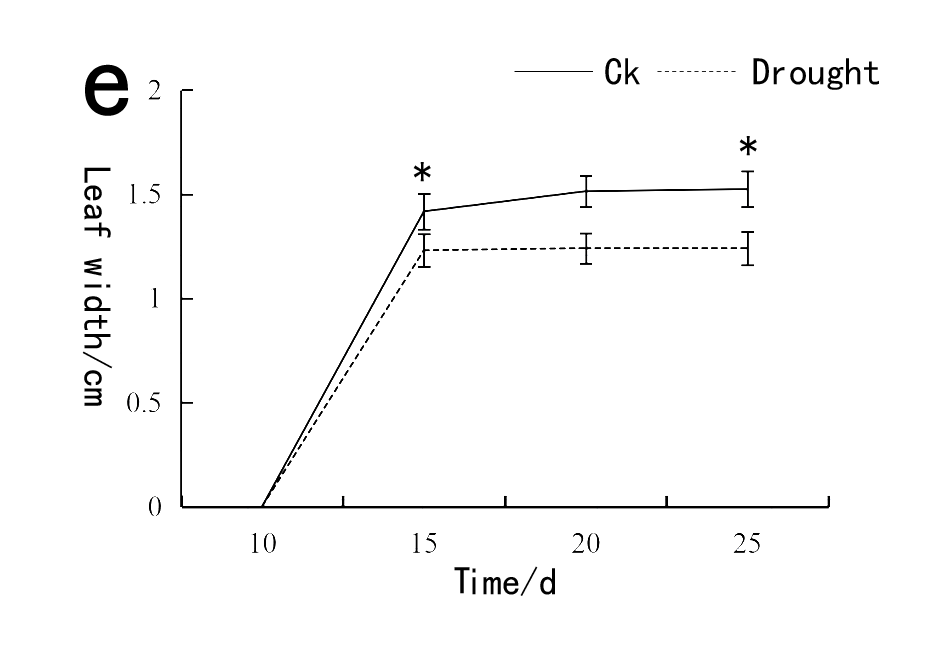

Supplement: Supplemental Information 2 [file peerj-11-16658-s002.zip › 7.27/Fig. 1/e.png]

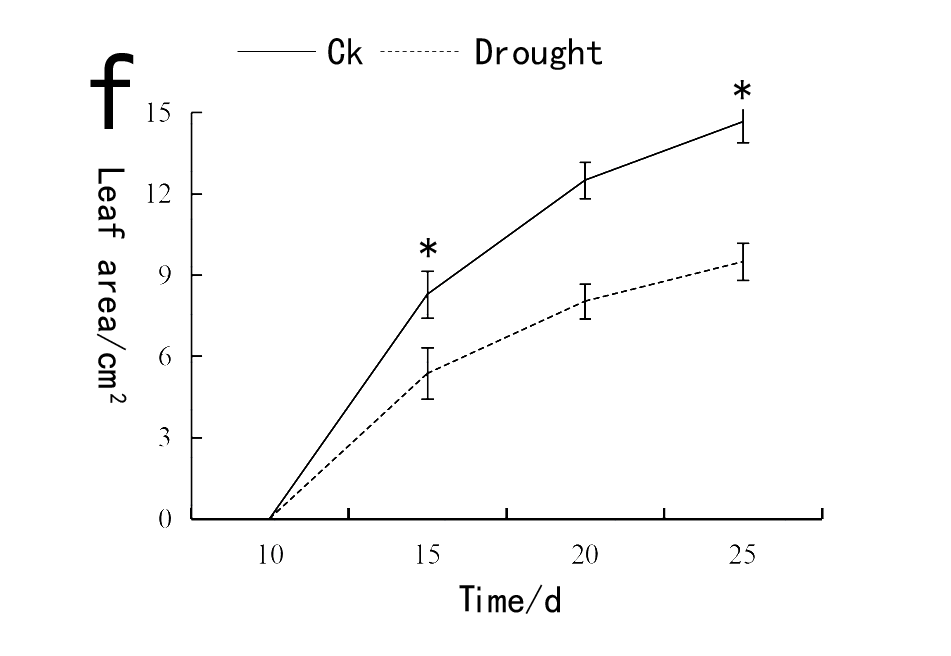

Supplement: Supplemental Information 2 [file peerj-11-16658-s002.zip › 7.27/Fig. 1/f.png]

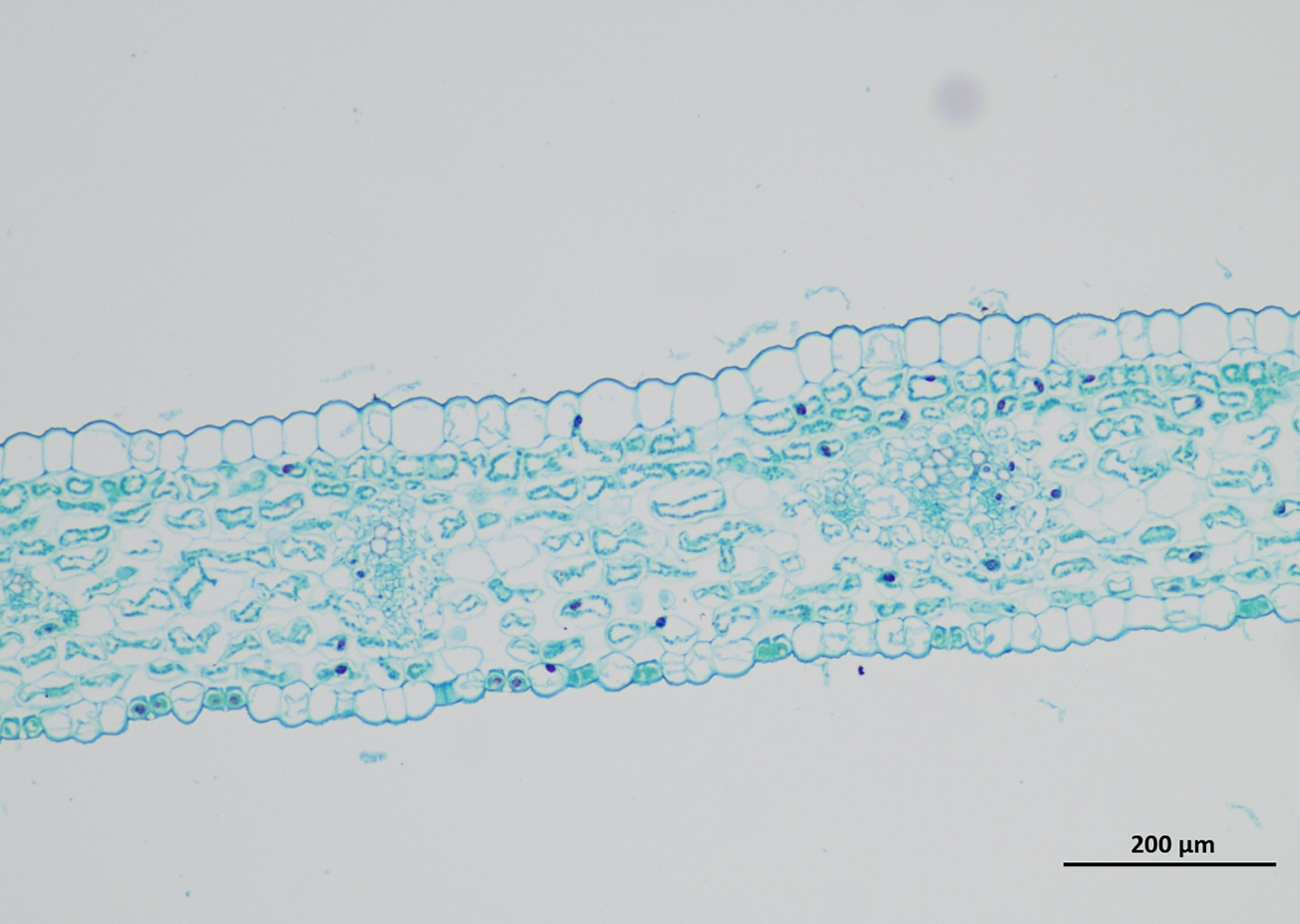

Supplement: Supplemental Information 2 [file peerj-11-16658-s002.zip › 7.27/Fig. 1/g.png]

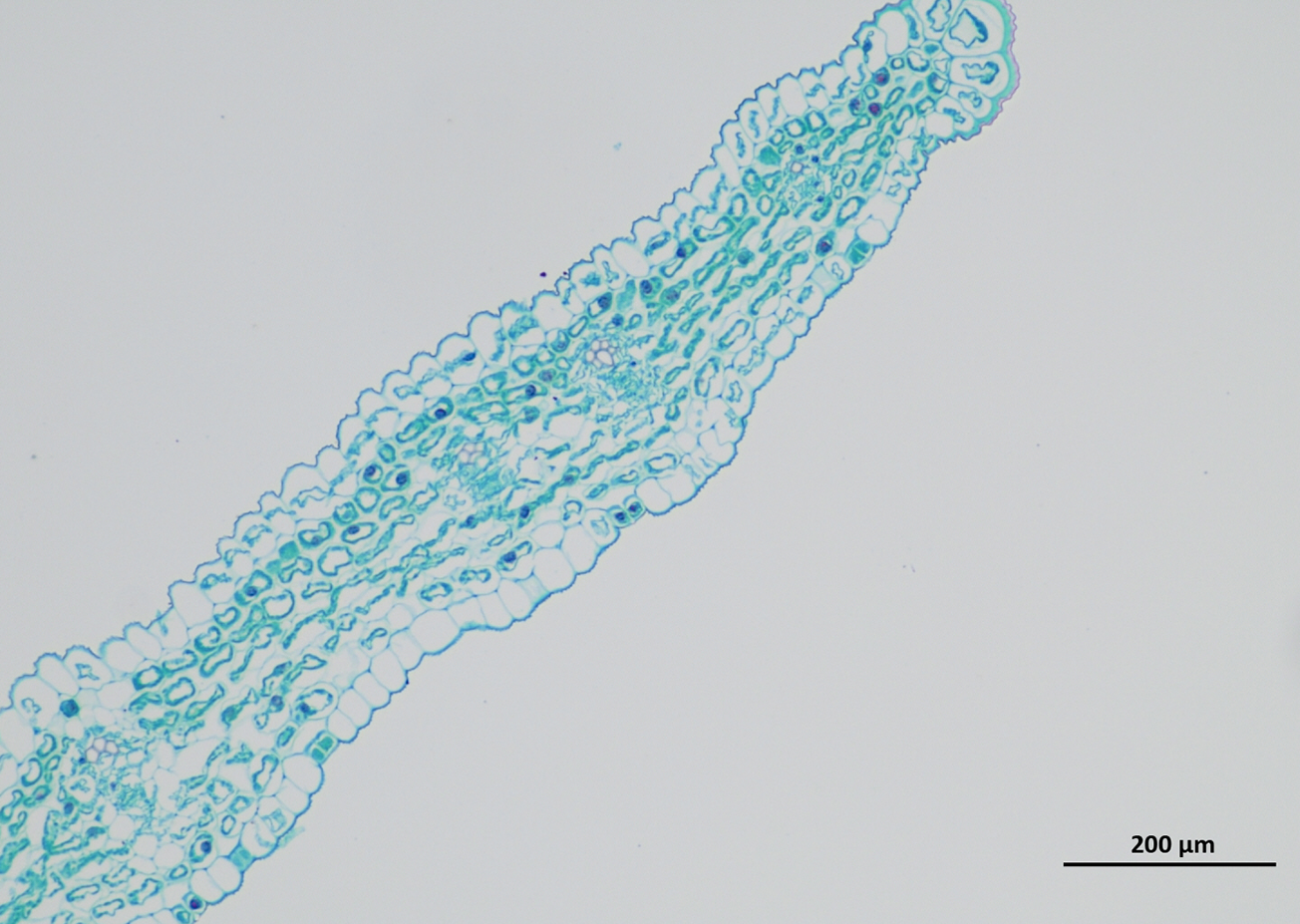

Supplement: Supplemental Information 2 [file peerj-11-16658-s002.zip › 7.27/Fig. 1/h.png]

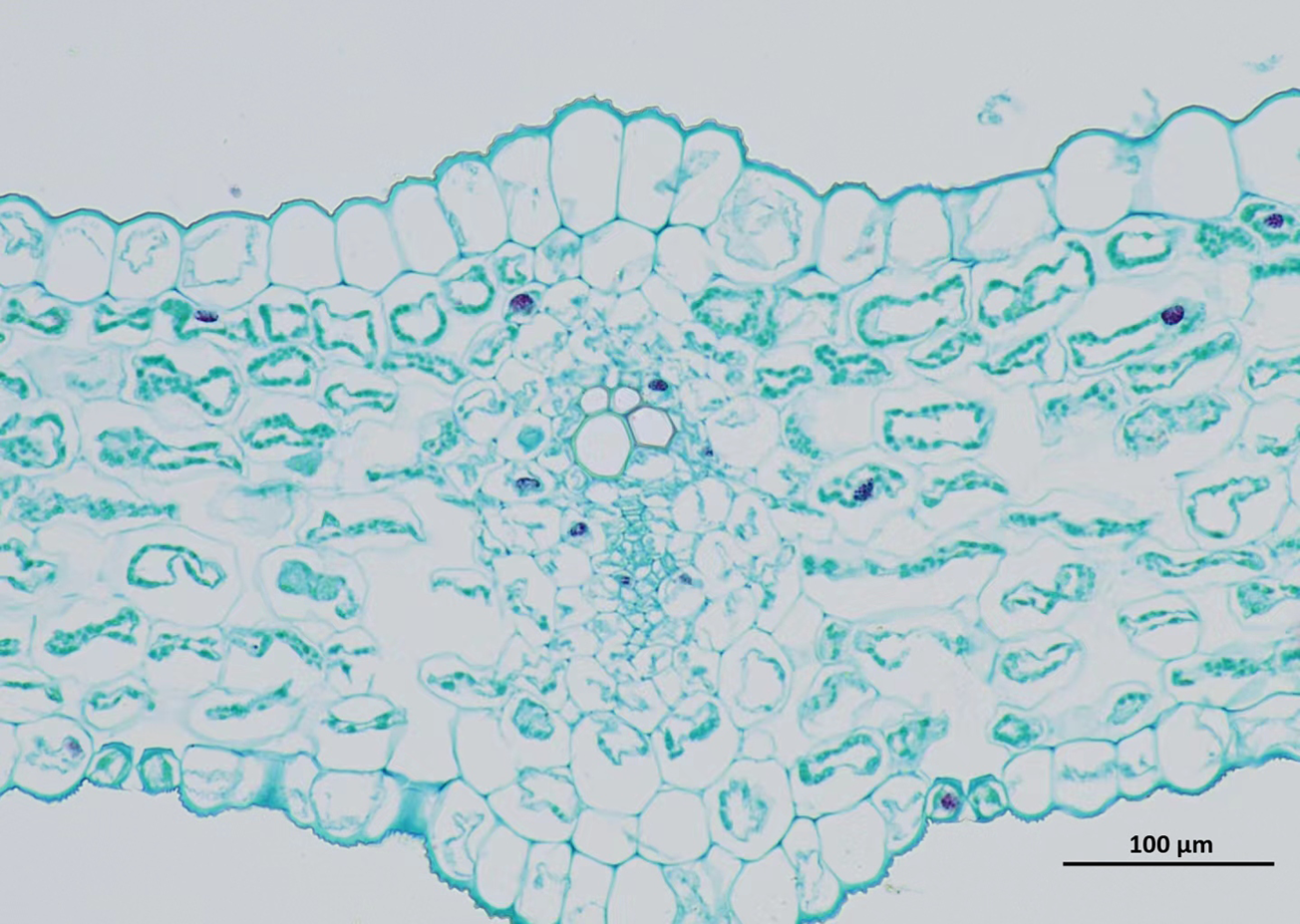

Supplement: Supplemental Information 2 [file peerj-11-16658-s002.zip › 7.27/Fig. 1/i.png]

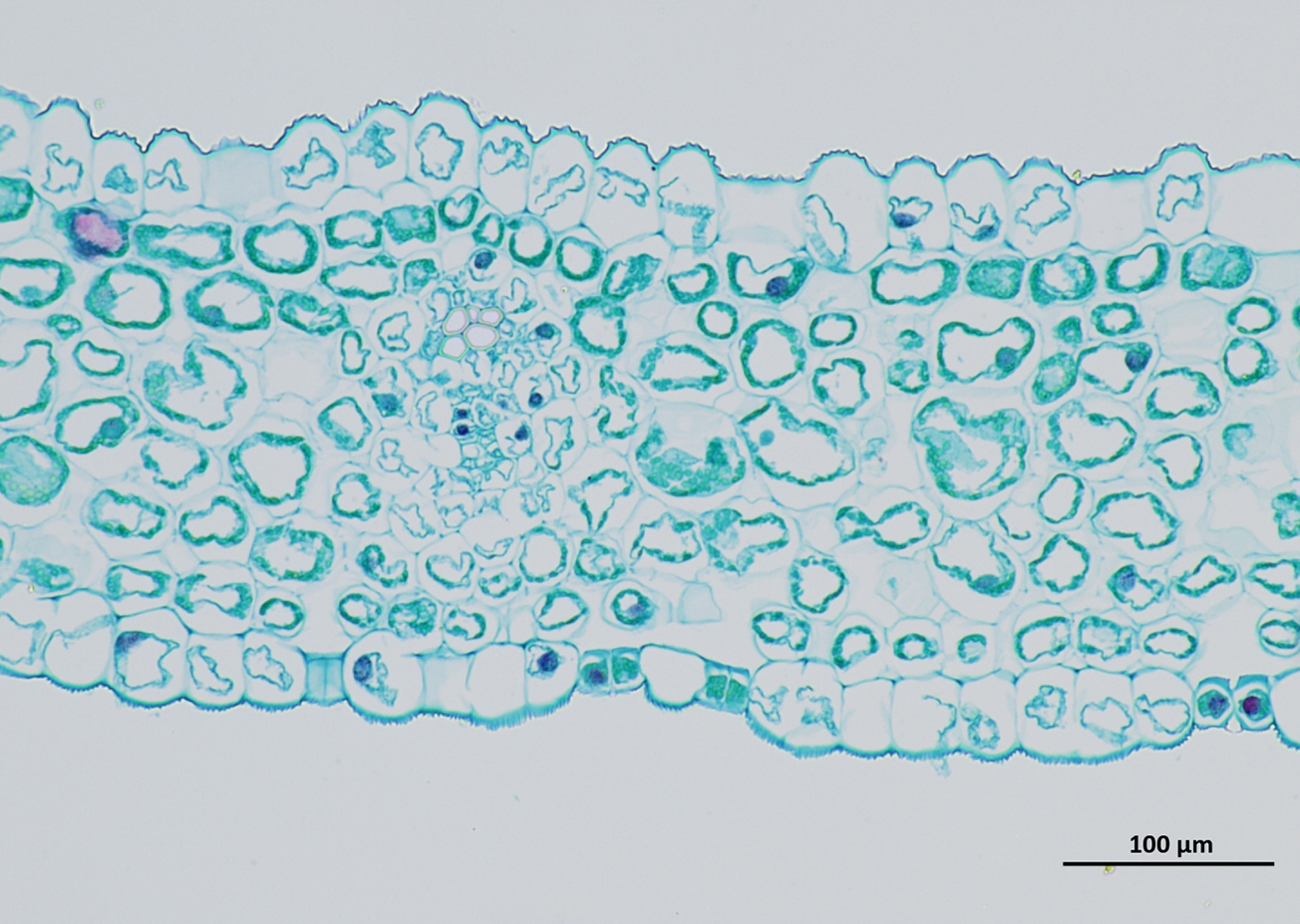

Supplement: Supplemental Information 2 [file peerj-11-16658-s002.zip › 7.27/Fig. 1/j.png]

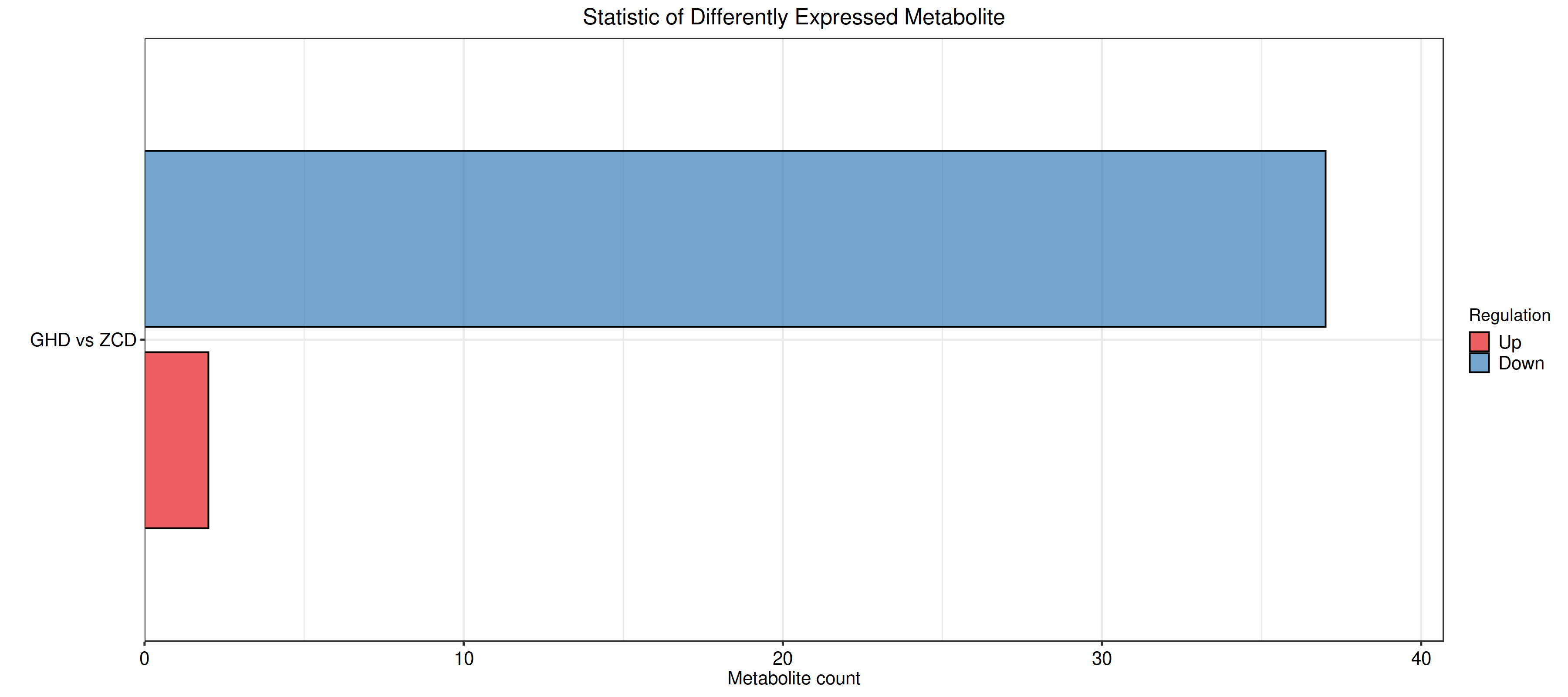

Supplement: Supplemental Information 2 [file peerj-11-16658-s002.zip › 7.27/Fig. 2/a.png]

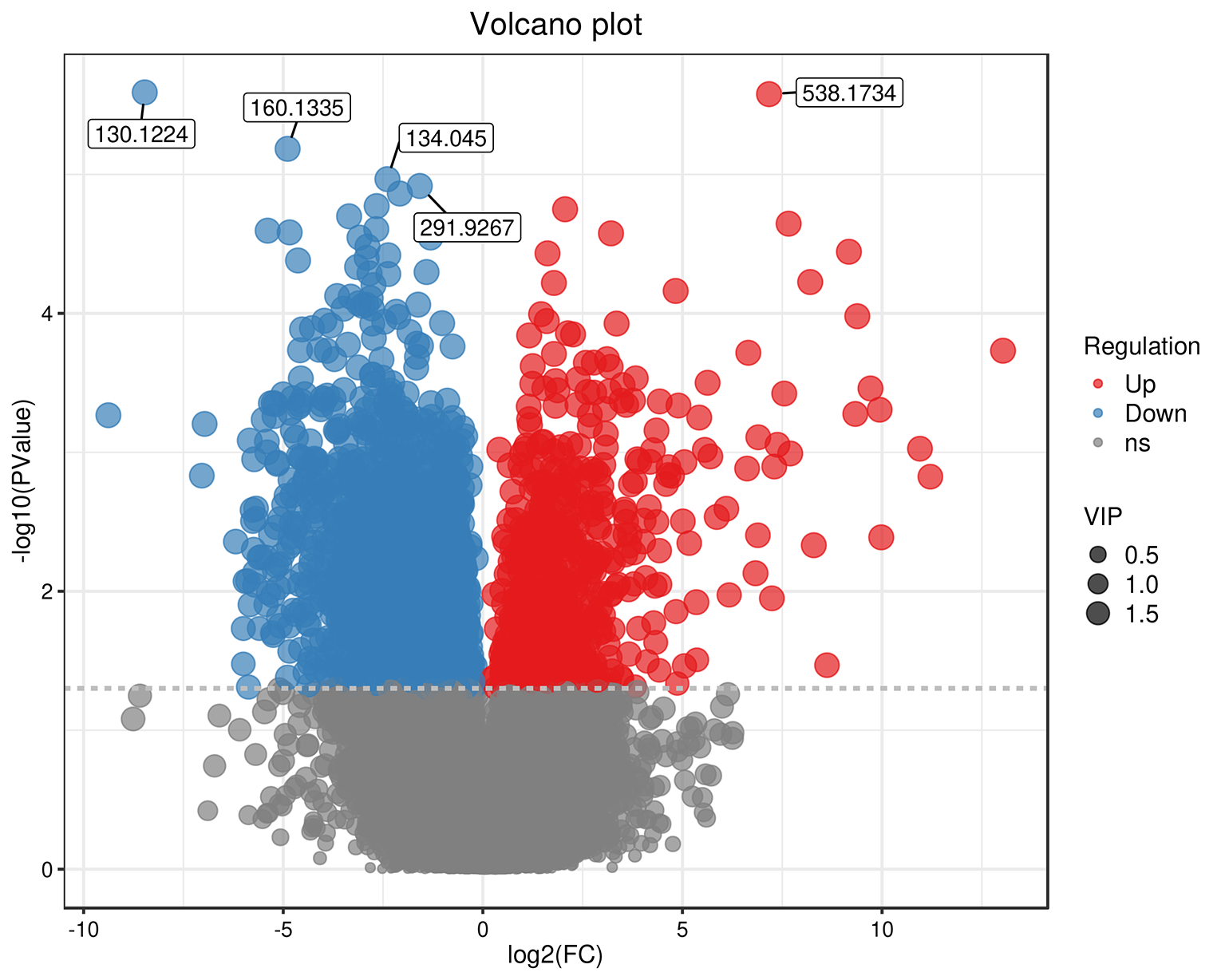

Supplement: Supplemental Information 2 [file peerj-11-16658-s002.zip › 7.27/Fig. 2/b.png]

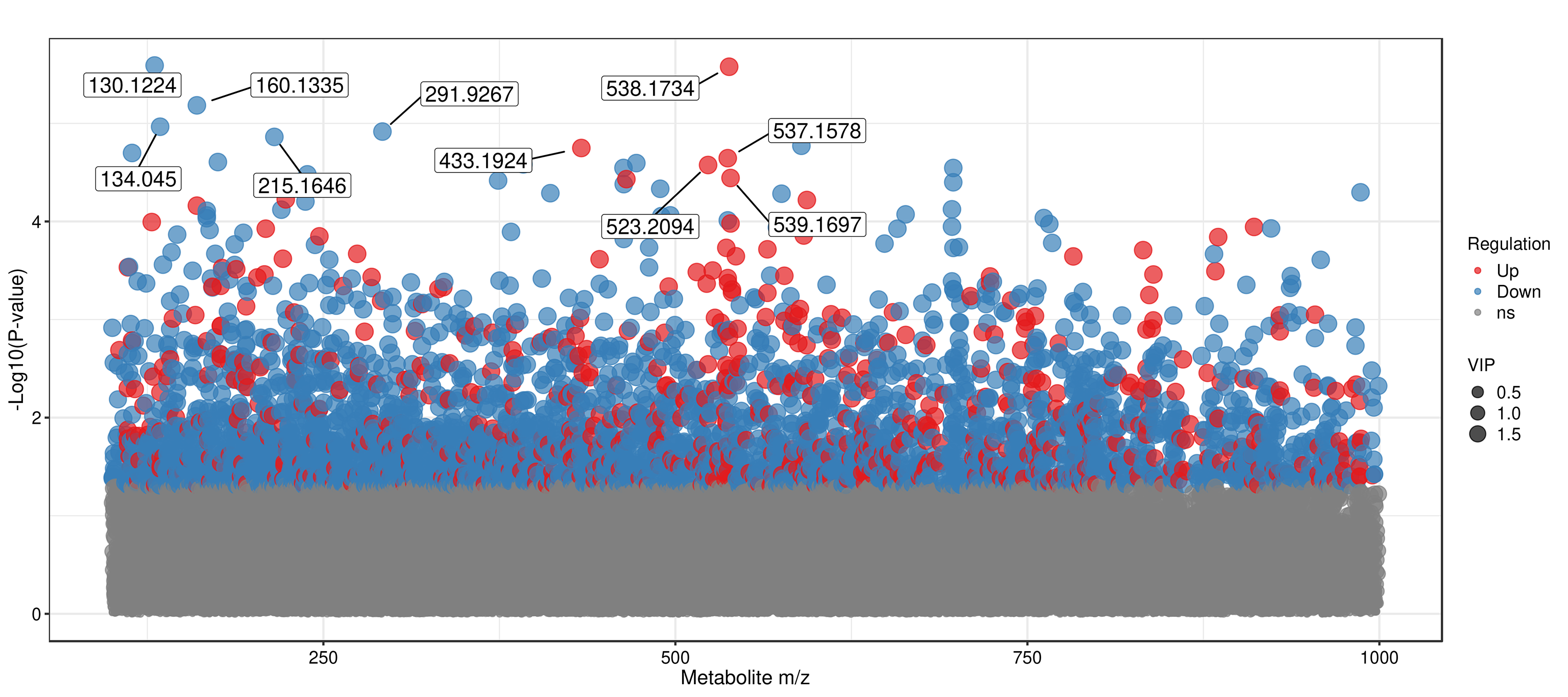

Supplement: Supplemental Information 2 [file peerj-11-16658-s002.zip › 7.27/Fig. 2/c.png]

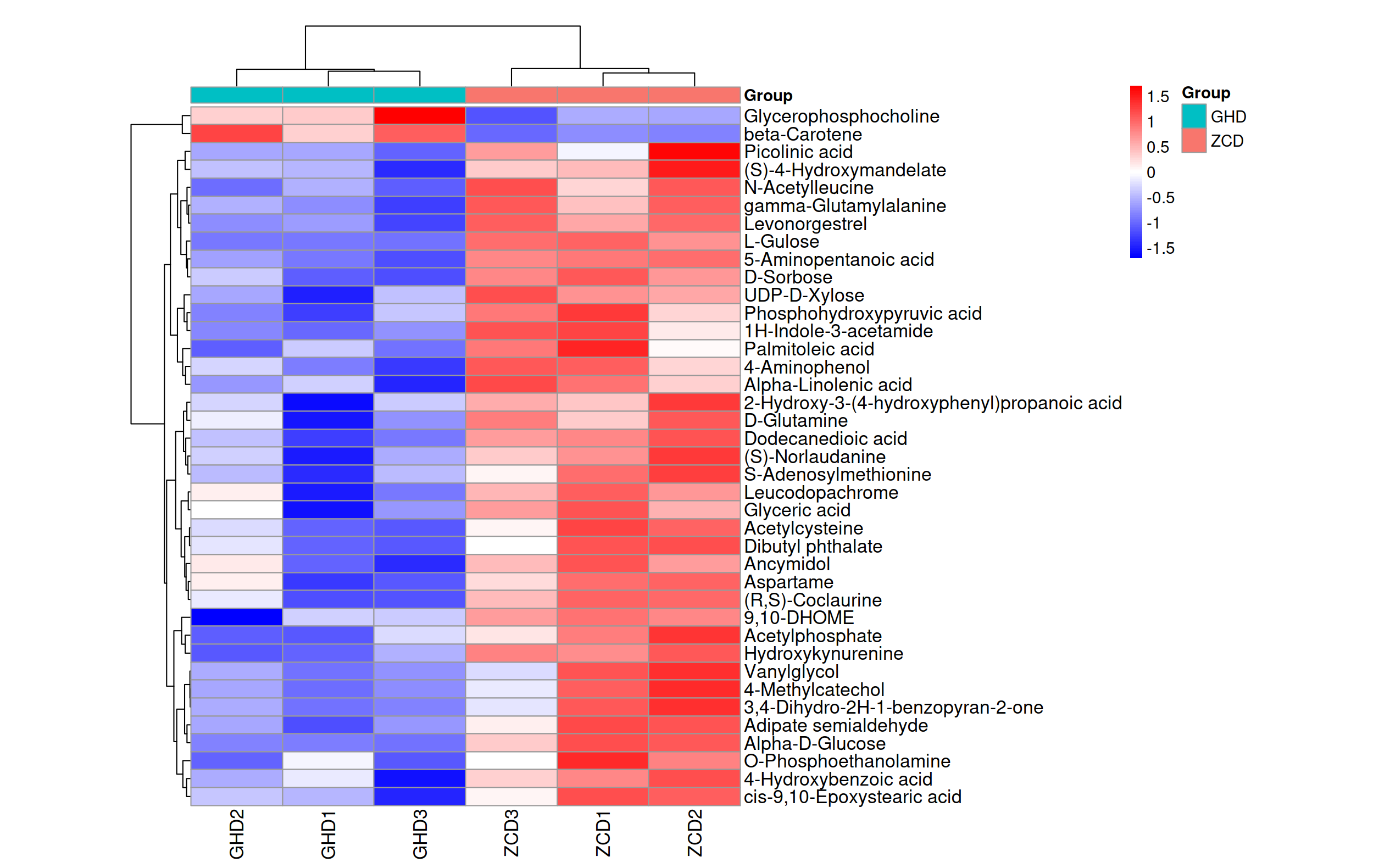

Supplement: Supplemental Information 2 [file peerj-11-16658-s002.zip › 7.27/Fig. 3/a.png]

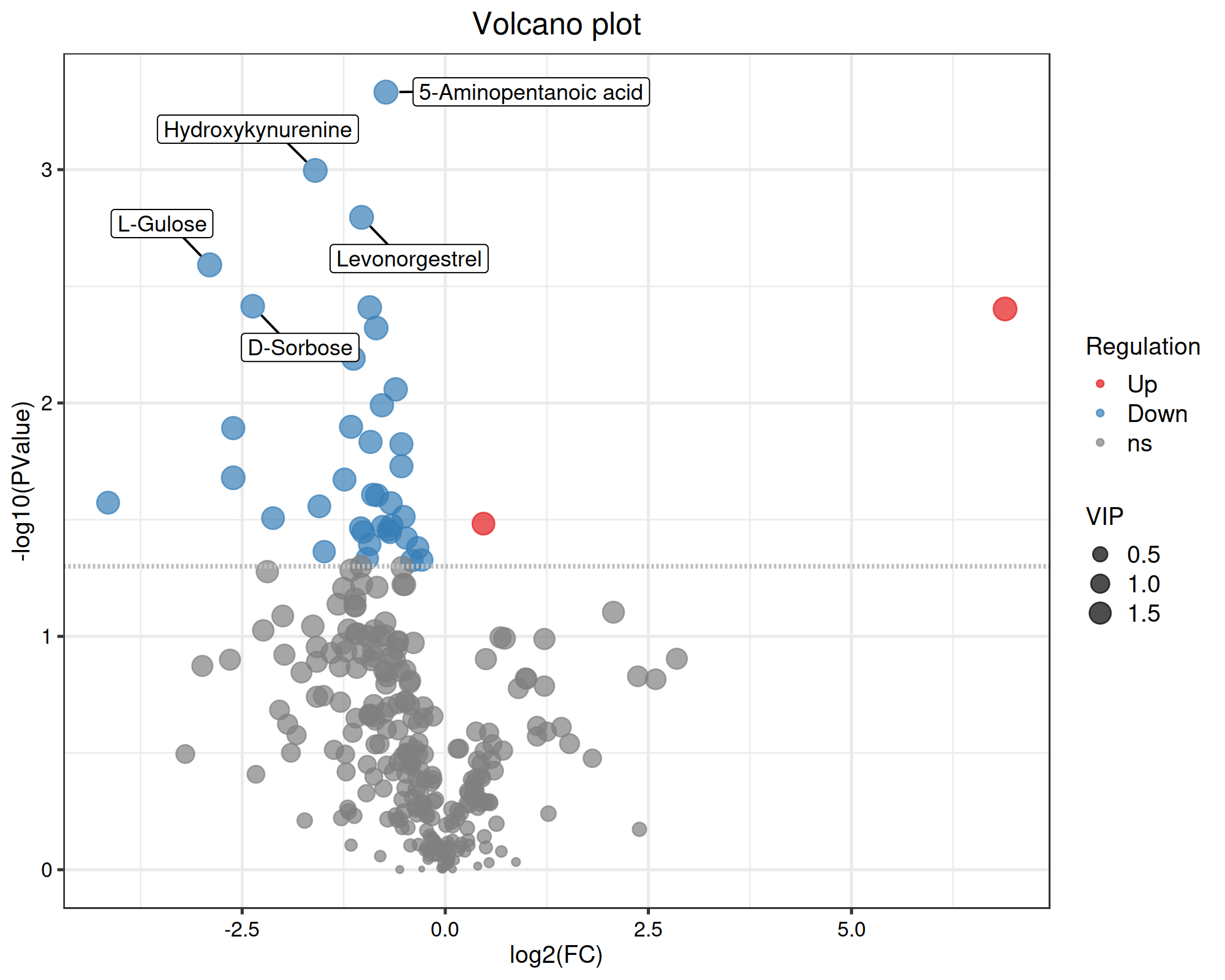

Supplement: Supplemental Information 2 [file peerj-11-16658-s002.zip › 7.27/Fig. 3/b.png]

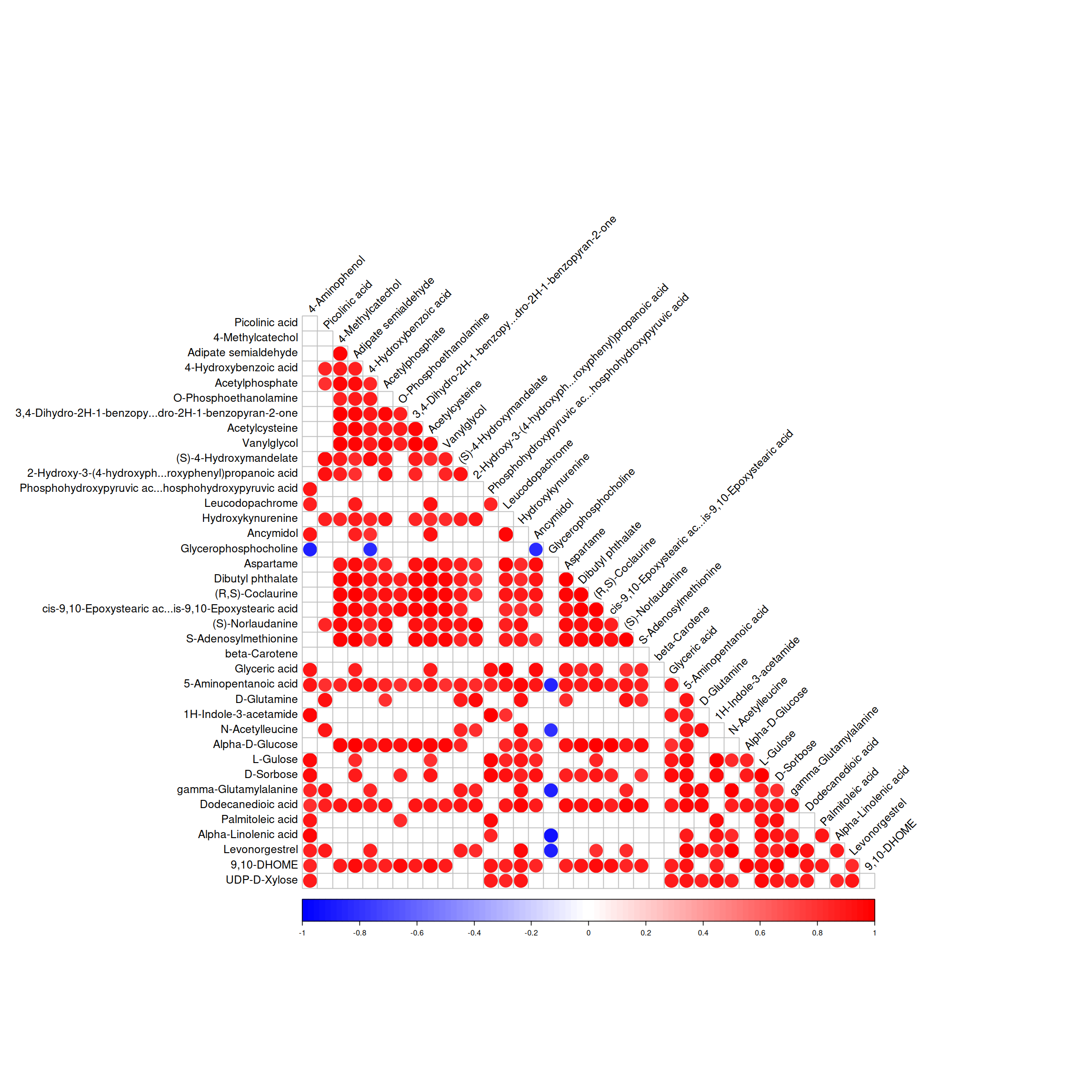

Supplement: Supplemental Information 2 [file peerj-11-16658-s002.zip › 7.27/Fig. 3/c.png]

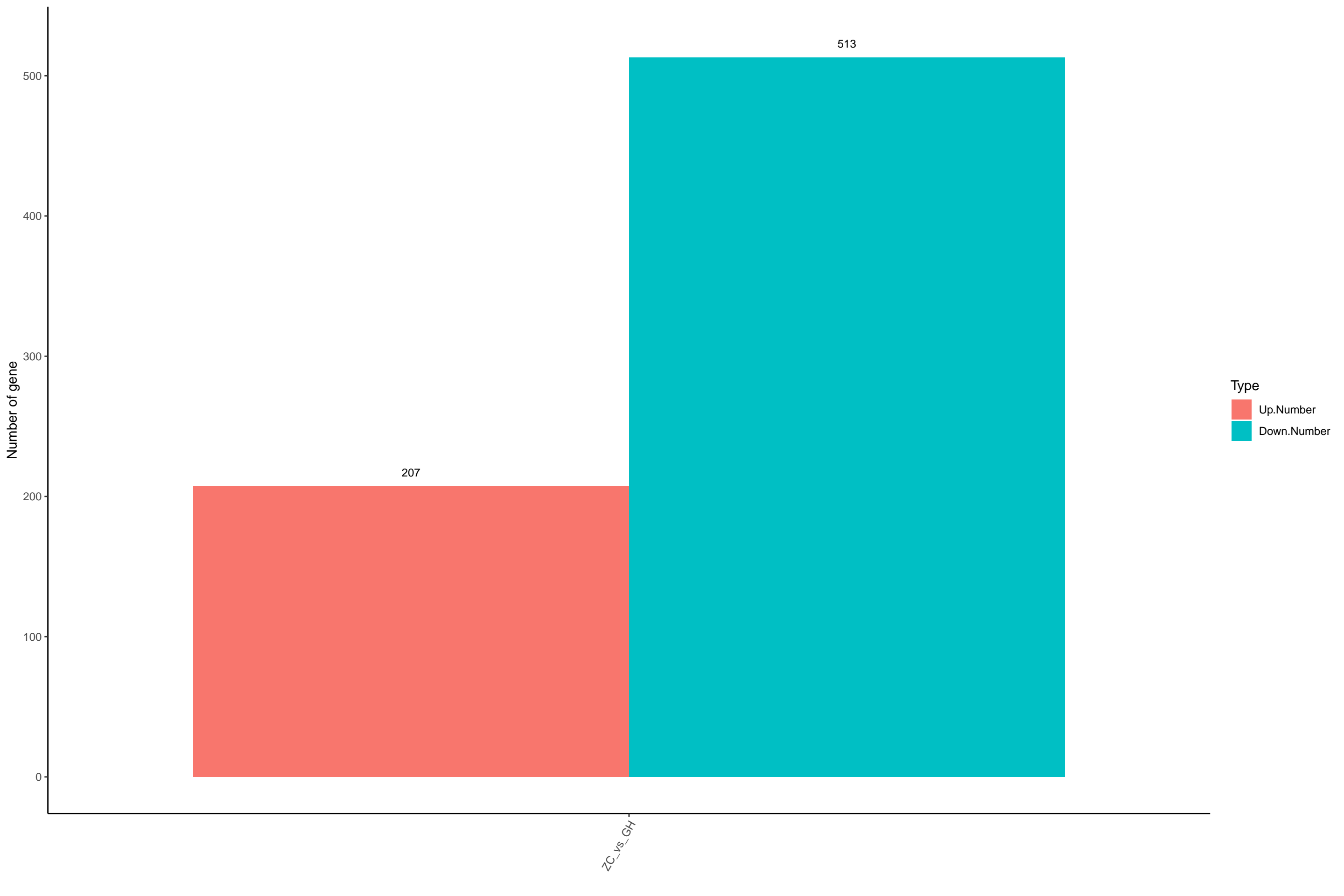

Supplement: Supplemental Information 2 [file peerj-11-16658-s002.zip › 7.27/Fig. 4/a.pdf]

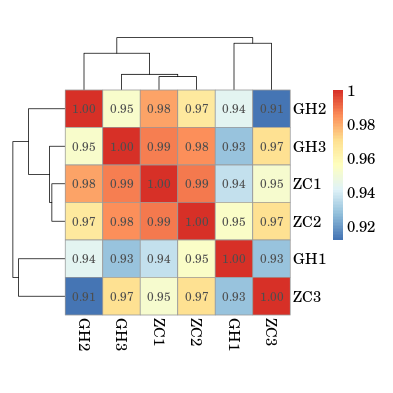

Supplement: Supplemental Information 2 [file peerj-11-16658-s002.zip › 7.27/Fig. 4/b.png]

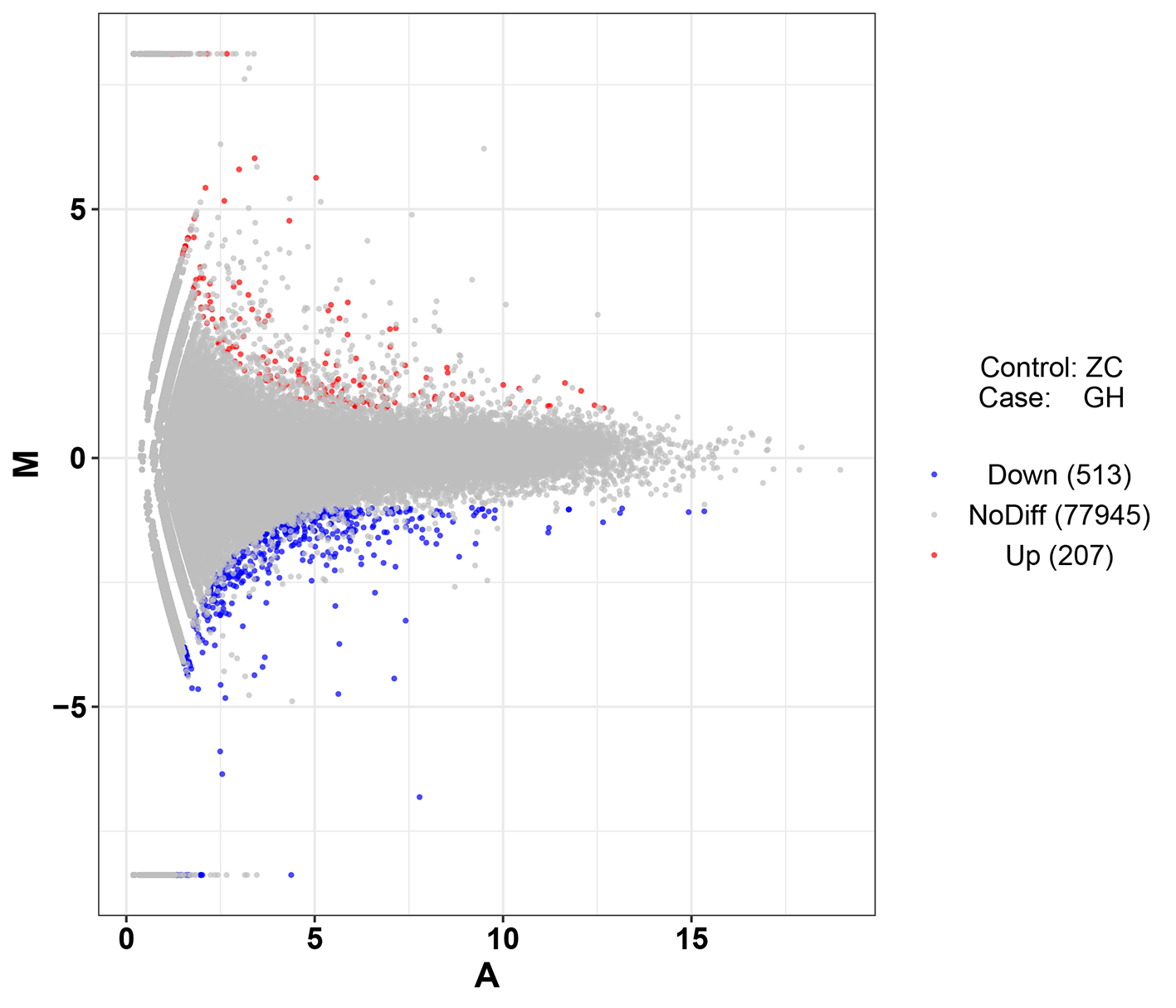

Supplement: Supplemental Information 2 [file peerj-11-16658-s002.zip › 7.27/Fig. 4/c.png]

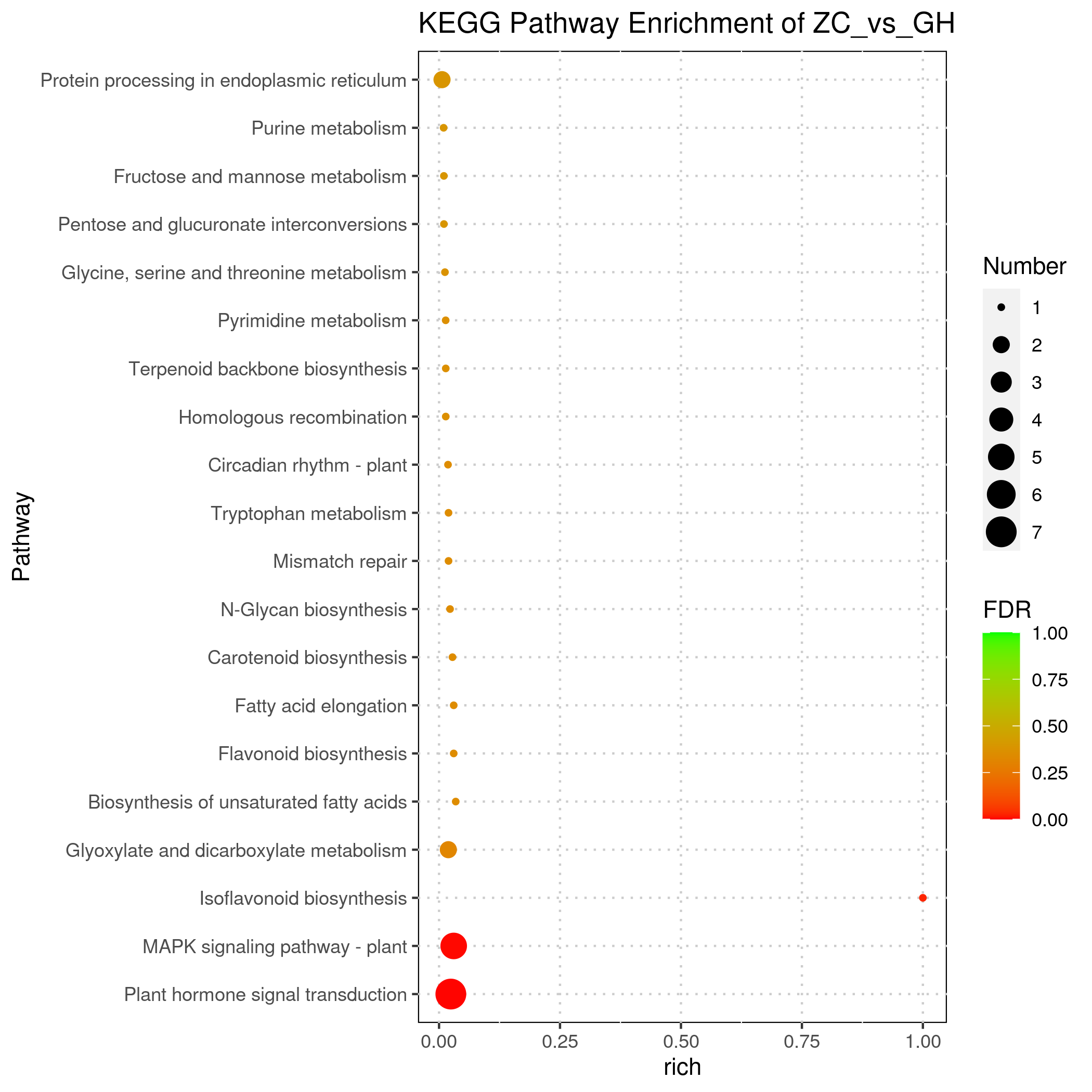

Supplement: Supplemental Information 2 [file peerj-11-16658-s002.zip › 7.27/Fig. 4/d.png]

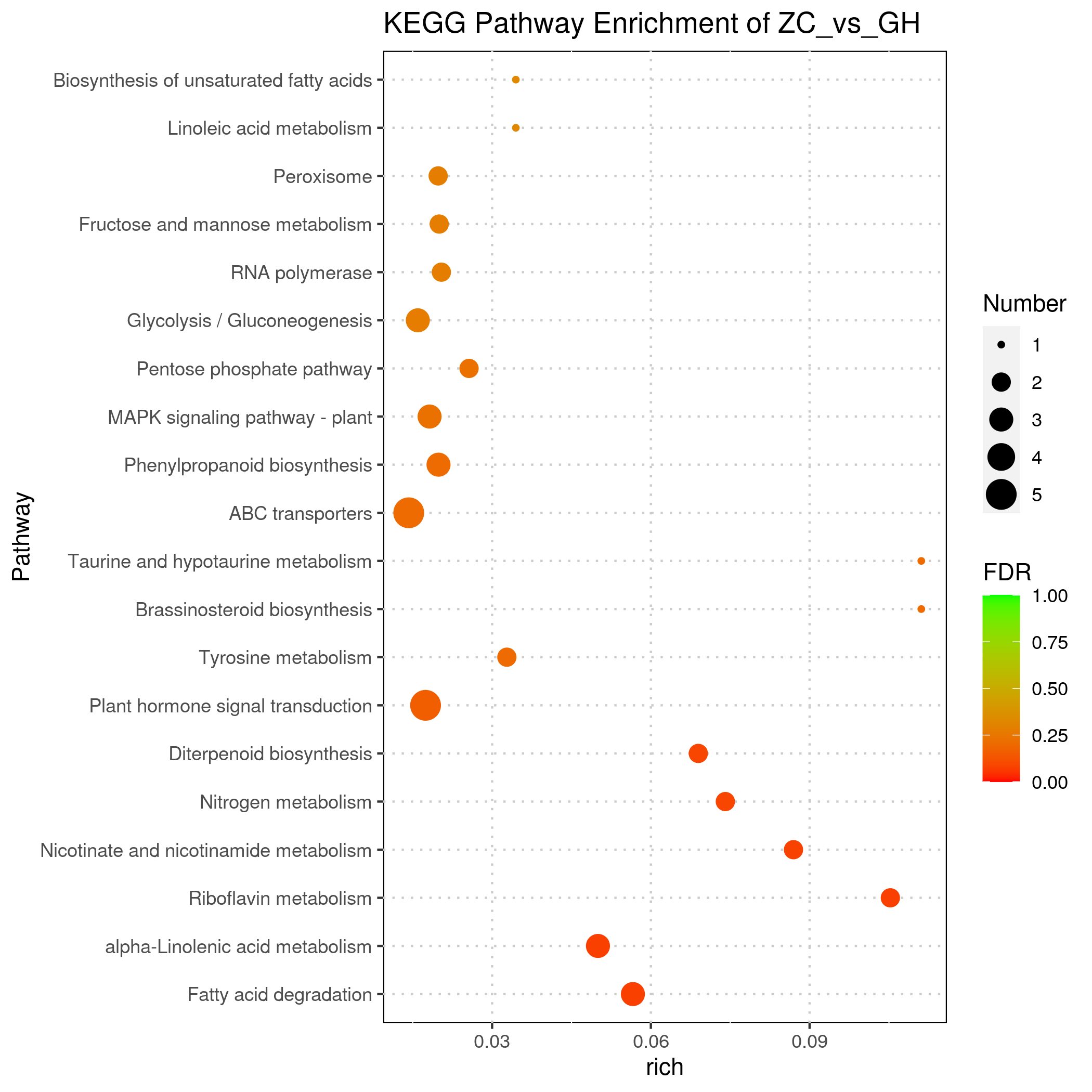

Supplement: Supplemental Information 2 [file peerj-11-16658-s002.zip › 7.27/Fig. 4/e.png]

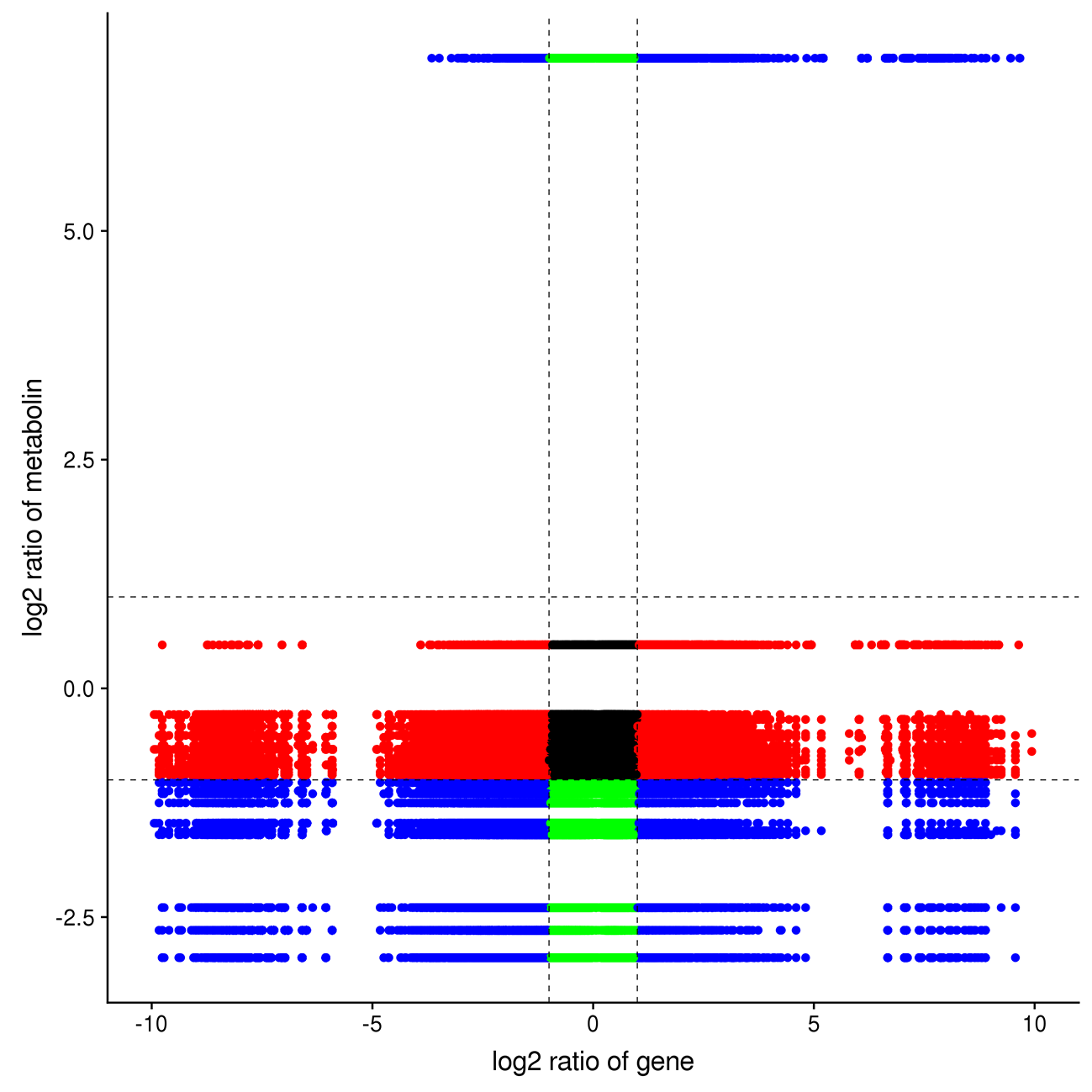

Supplement: Supplemental Information 2 [file peerj-11-16658-s002.zip › 7.27/Fig. 5/a.png]

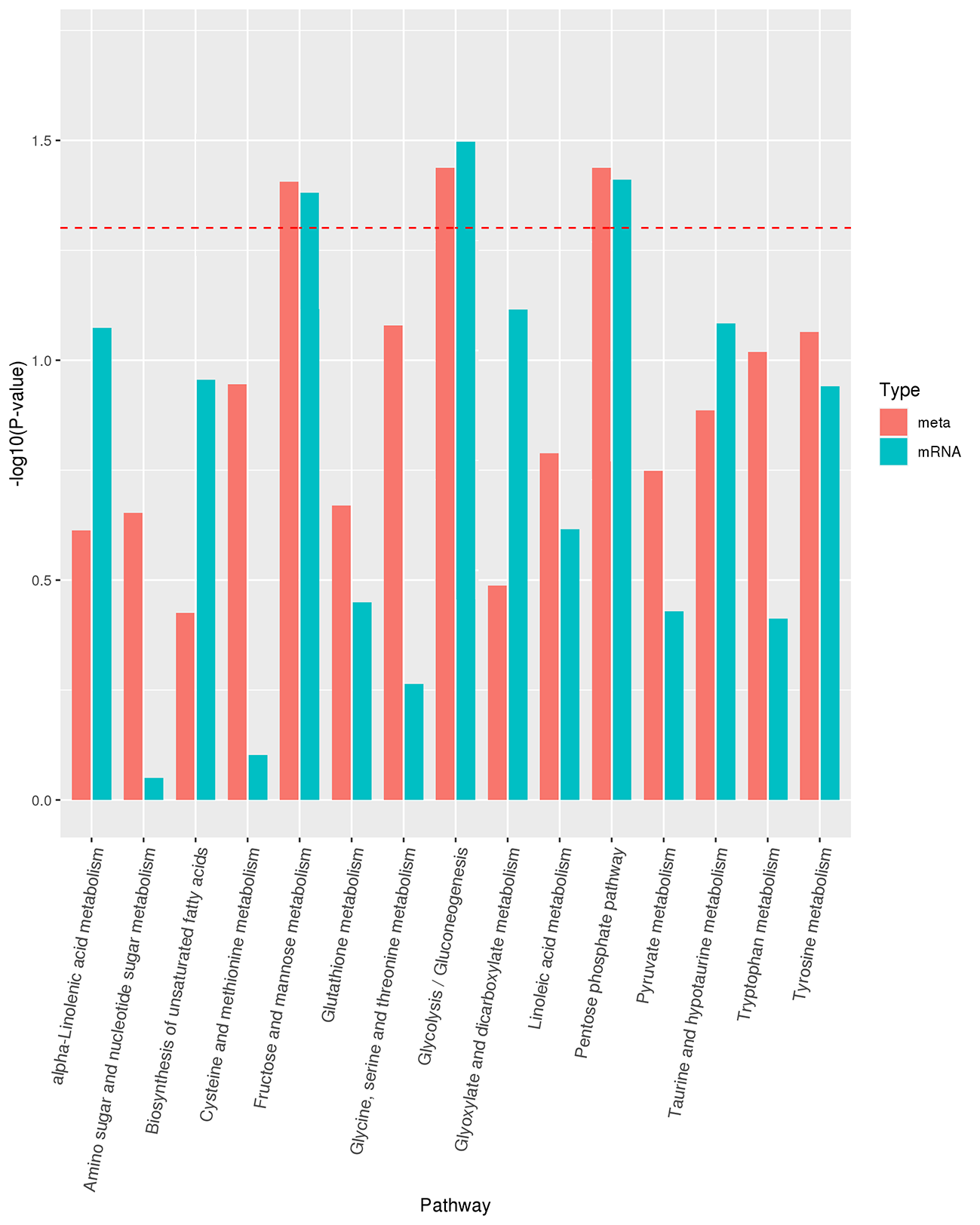

Supplement: Supplemental Information 2 [file peerj-11-16658-s002.zip › 7.27/Fig. 5/b.png]

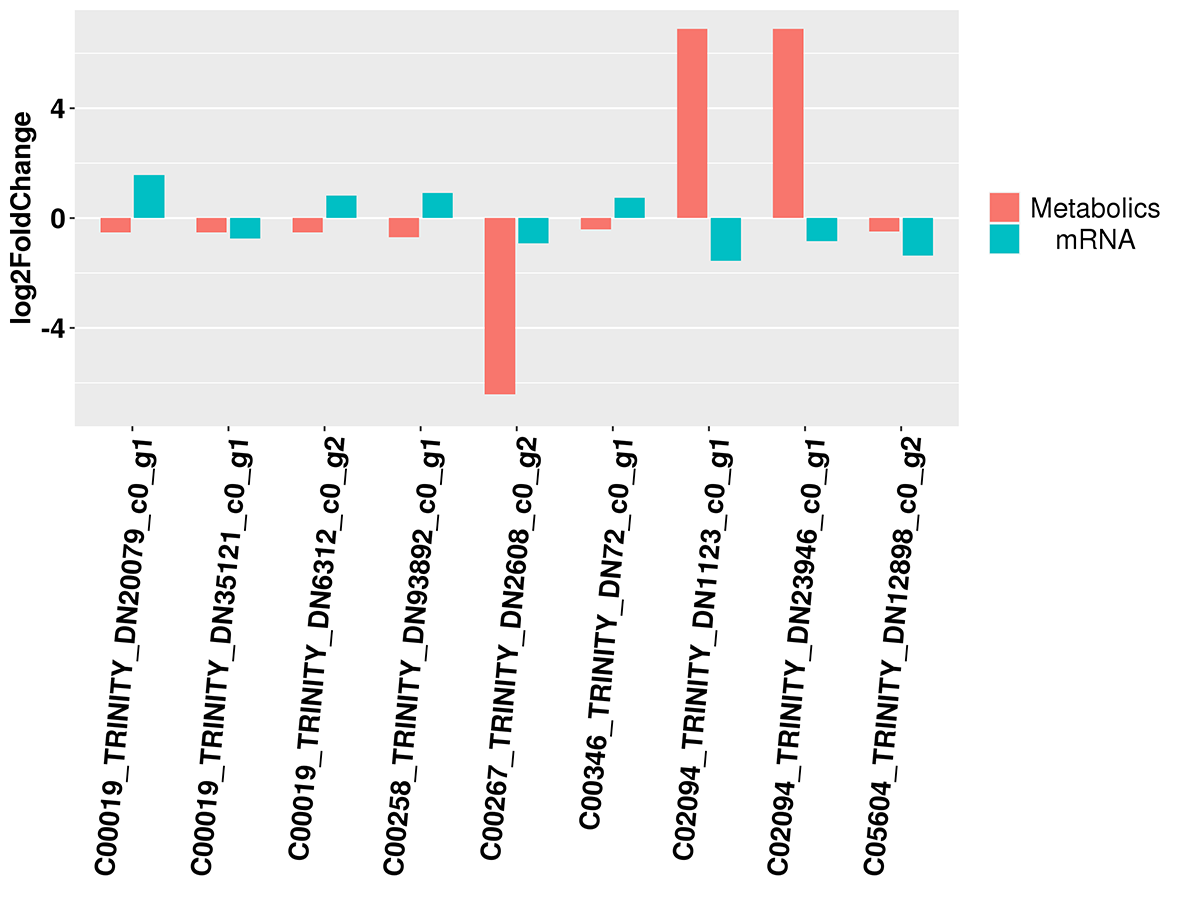

Supplement: Supplemental Information 2 [file peerj-11-16658-s002.zip › 7.27/Fig. 5/c.png]

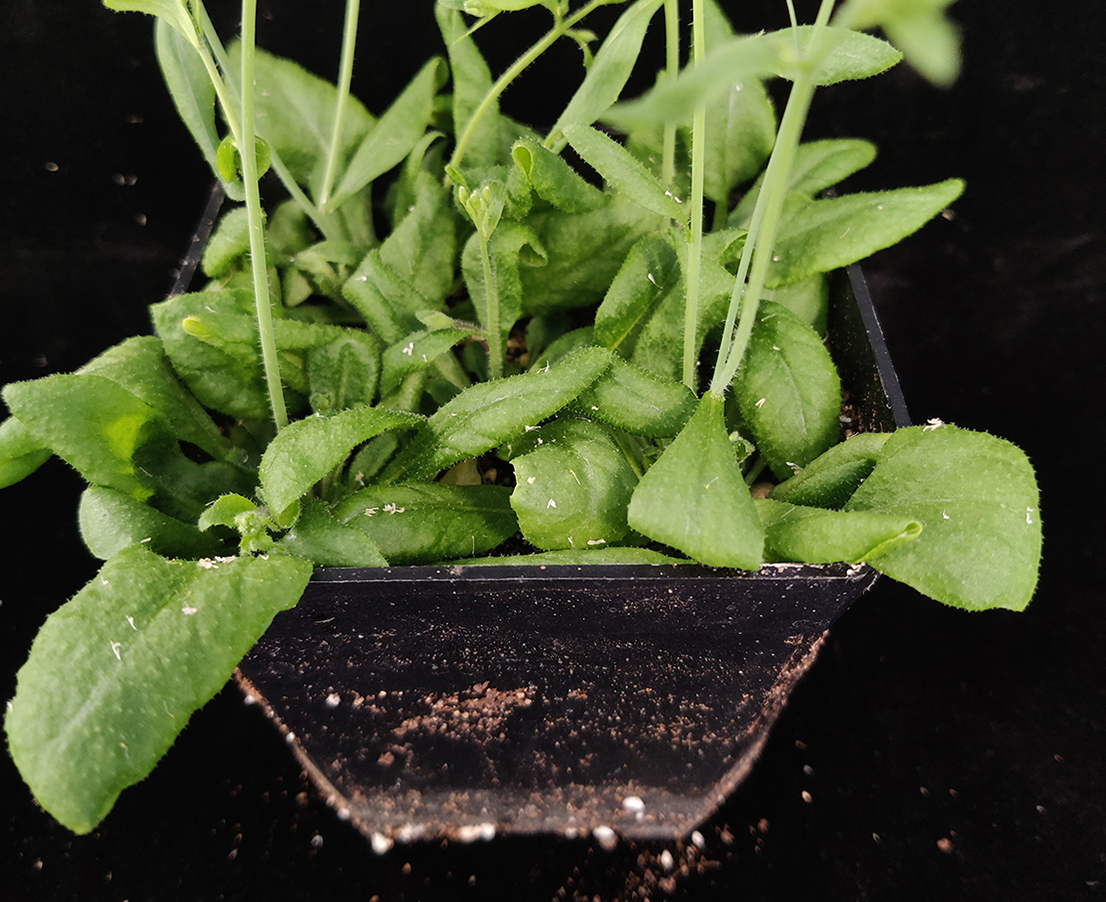

Supplement: Supplemental Information 2 [file peerj-11-16658-s002.zip › 7.27/Fig. 6/OE-2.png]

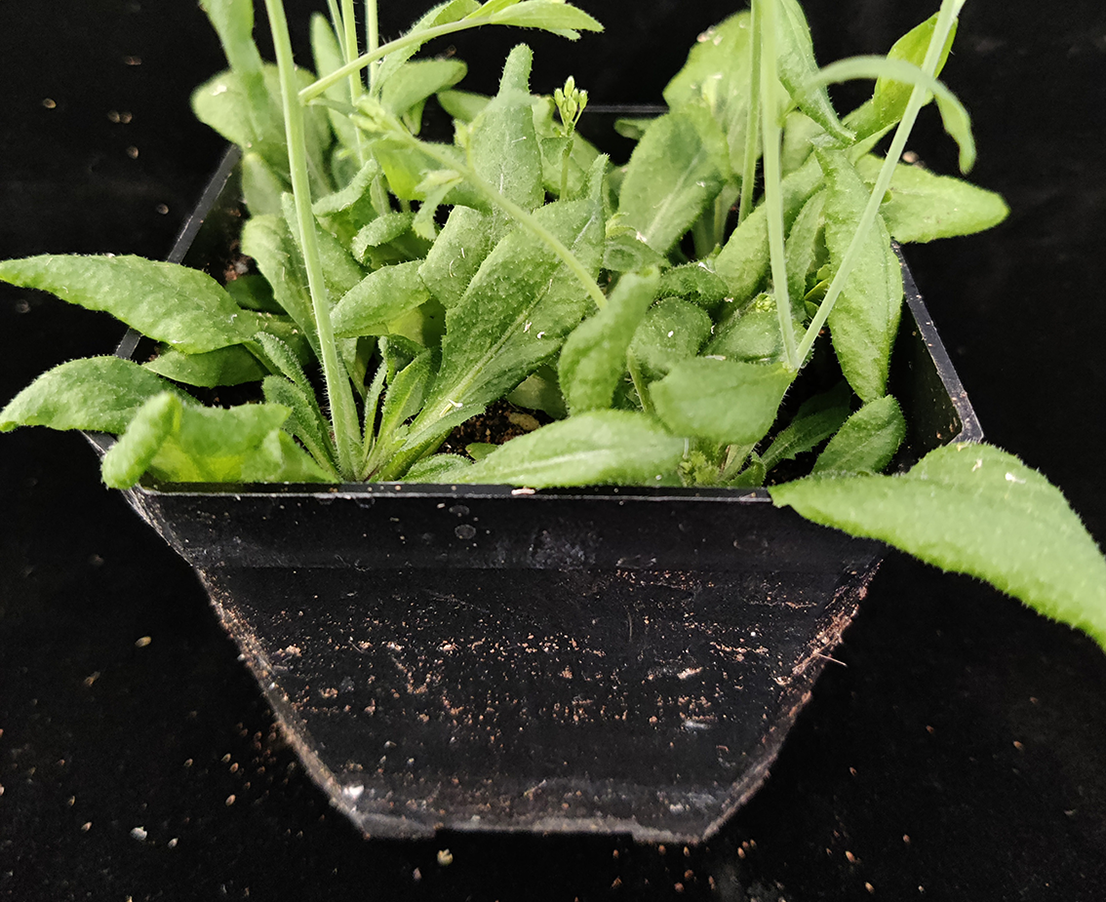

Supplement: Supplemental Information 2 [file peerj-11-16658-s002.zip › 7.27/Fig. 6/OE-3.png]

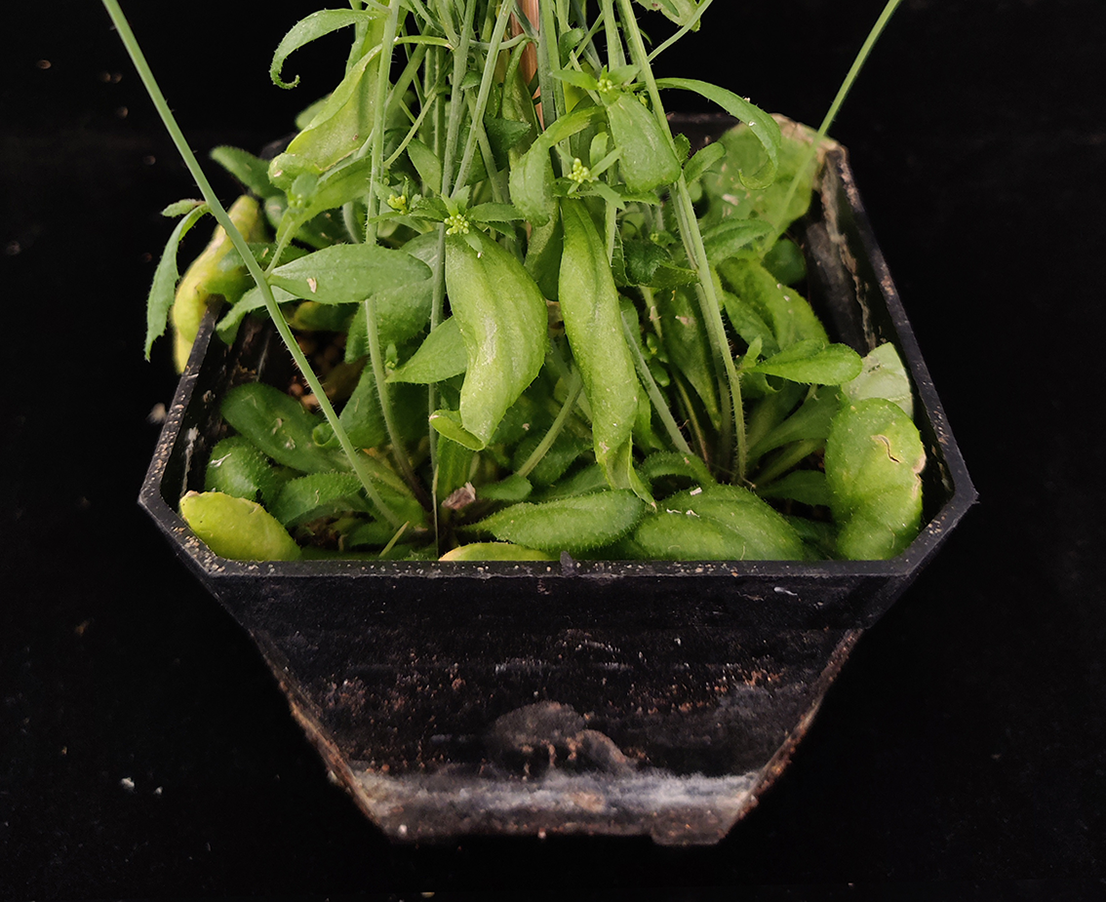

Supplement: Supplemental Information 2 [file peerj-11-16658-s002.zip › 7.27/Fig. 6/OE-4.png]

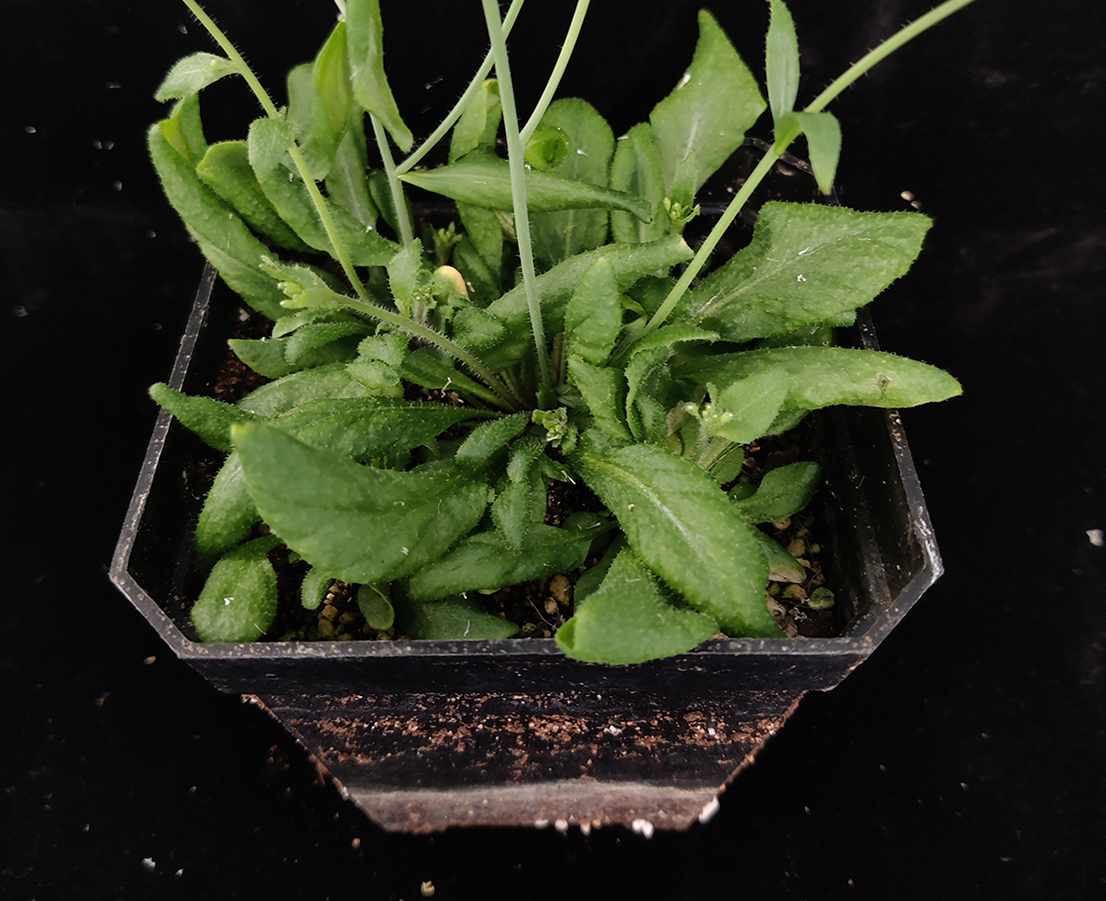

Supplement: Supplemental Information 2 [file peerj-11-16658-s002.zip › 7.27/Fig. 6/WT.png]

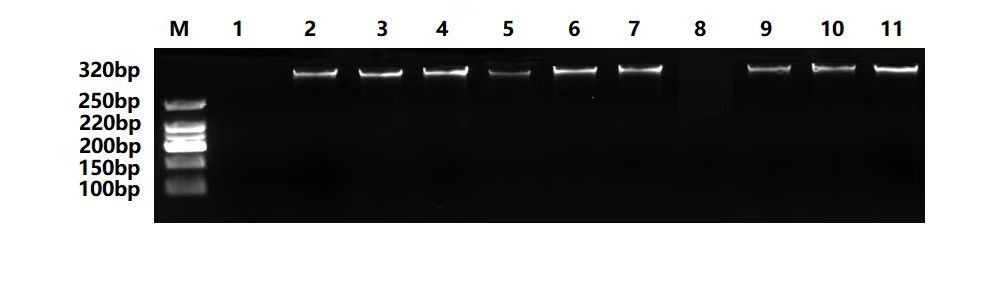

Supplement: Supplemental Information 2 [file peerj-11-16658-s002.zip › 7.27/Fig. 6/a.png]

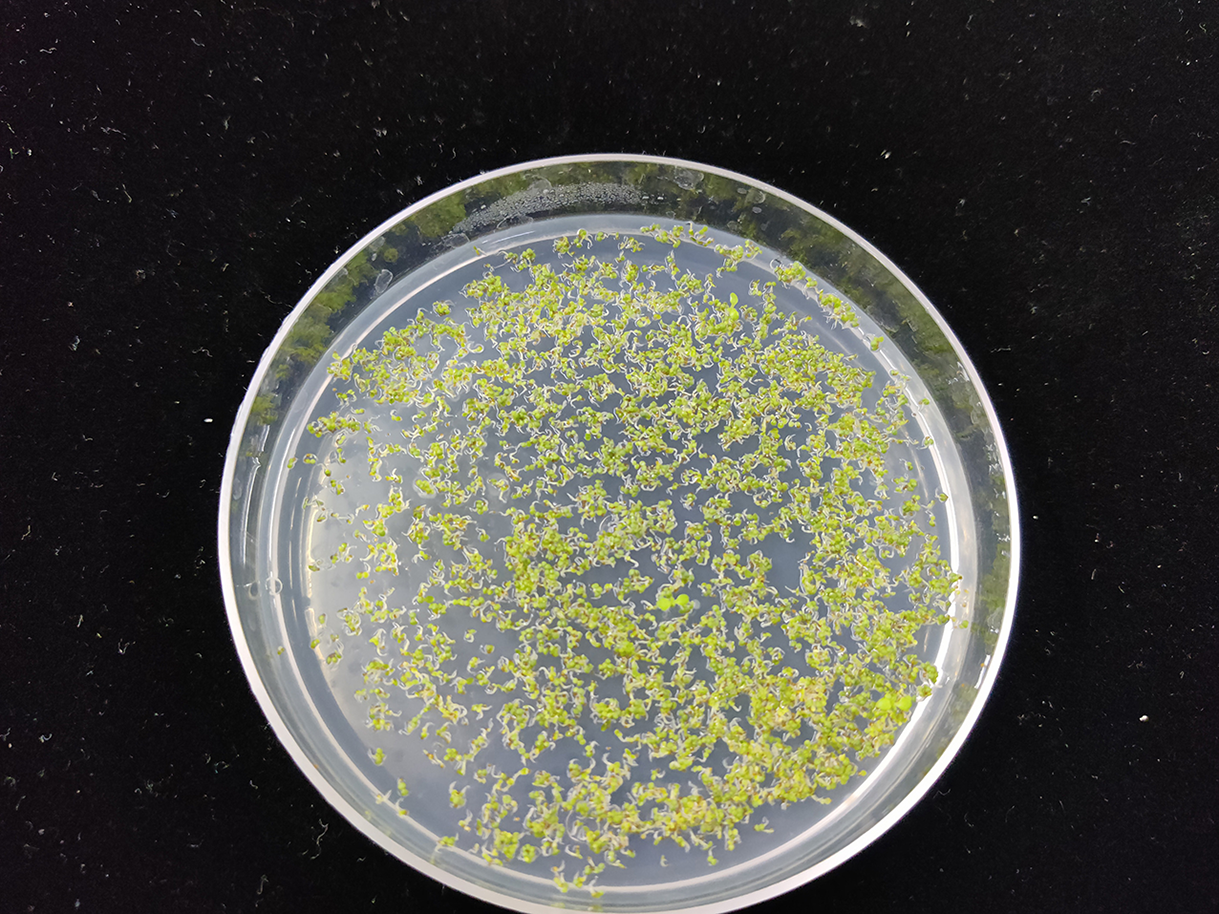

Supplement: Supplemental Information 2 [file peerj-11-16658-s002.zip › 7.27/Fig. 6/b.png]

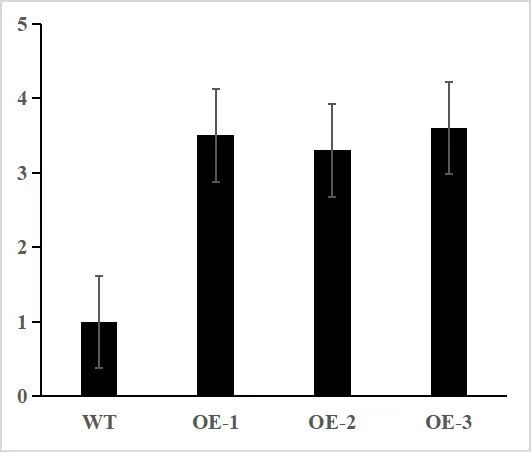

Supplement: Supplemental Information 2 [file peerj-11-16658-s002.zip › 7.27/Fig. 6/c.png]

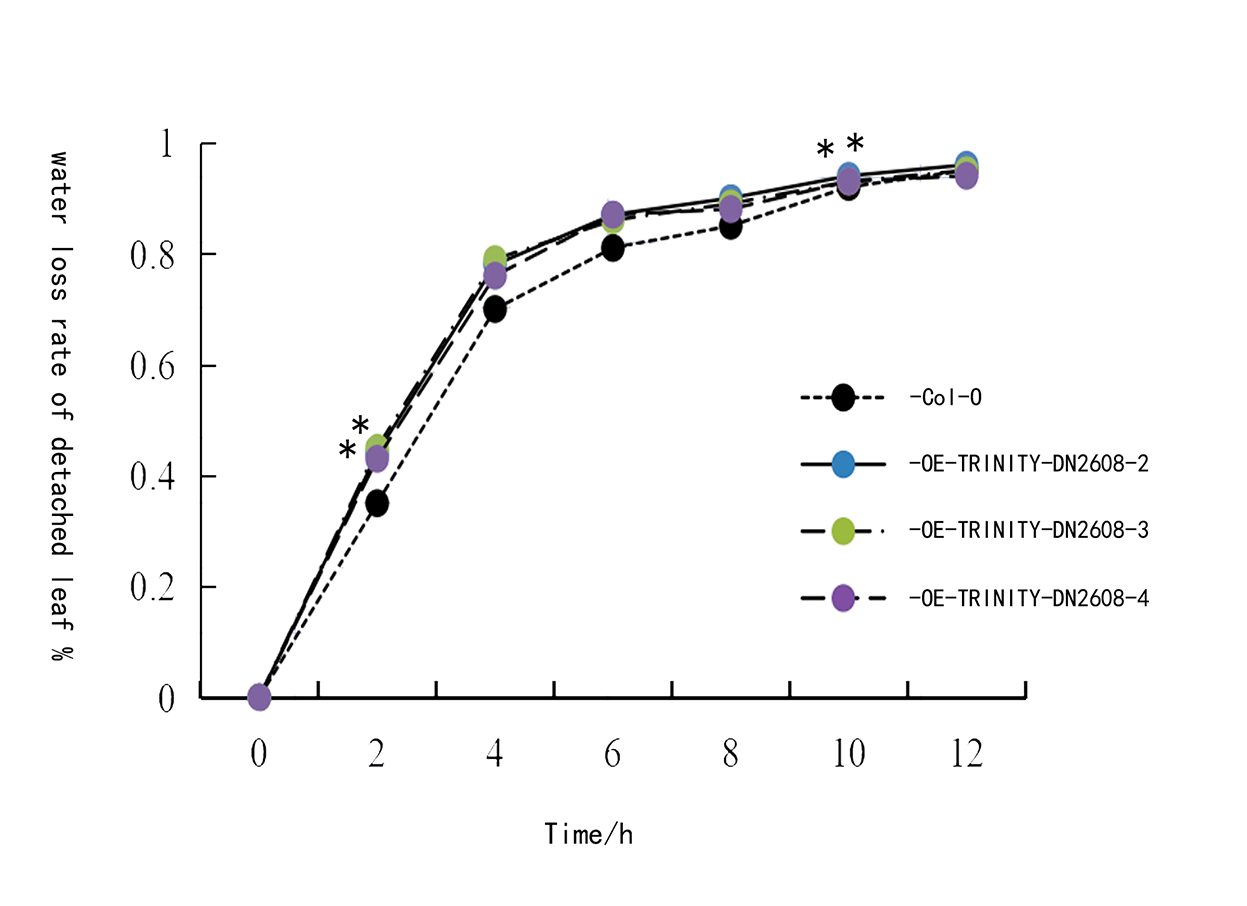

Supplement: Supplemental Information 2 [file peerj-11-16658-s002.zip › 7.27/Fig. 6/f.png]

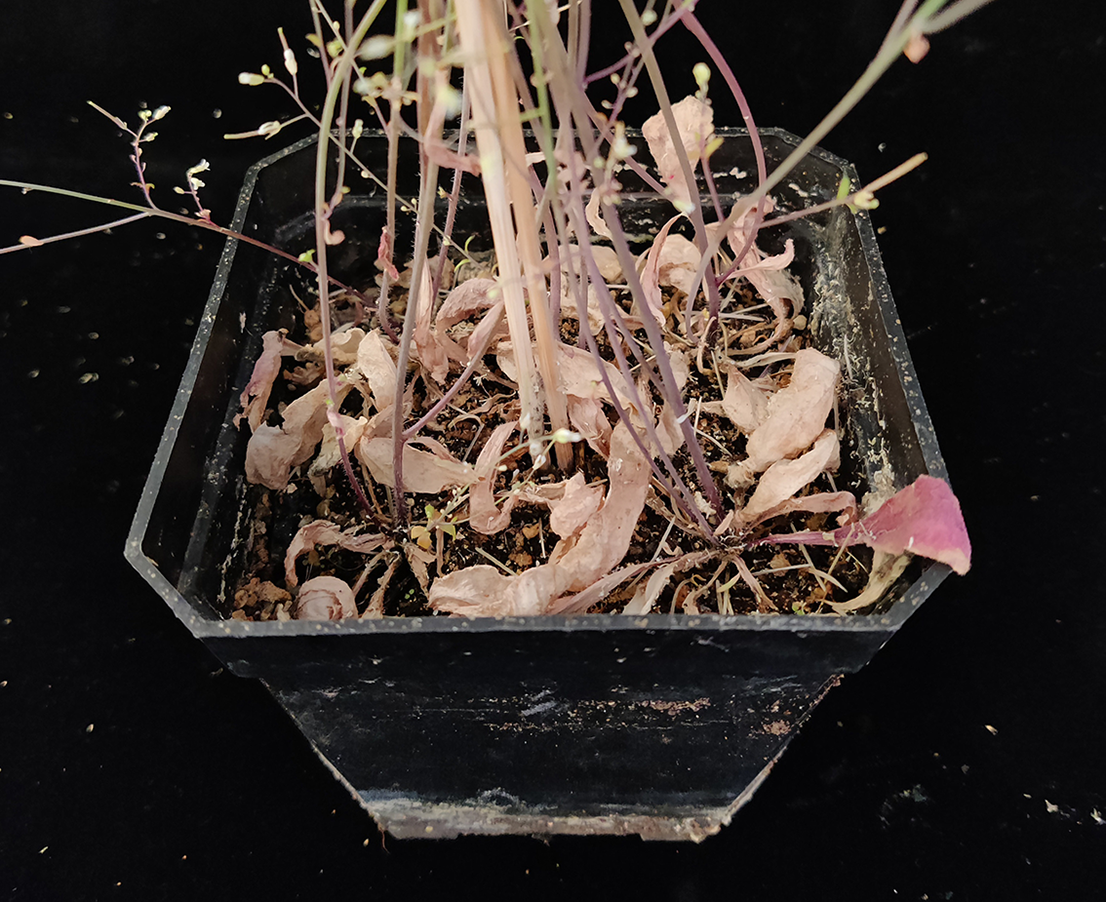

Supplement: Supplemental Information 2 [file peerj-11-16658-s002.zip › 7.27/Fig. 6/oe-2 (1).png]

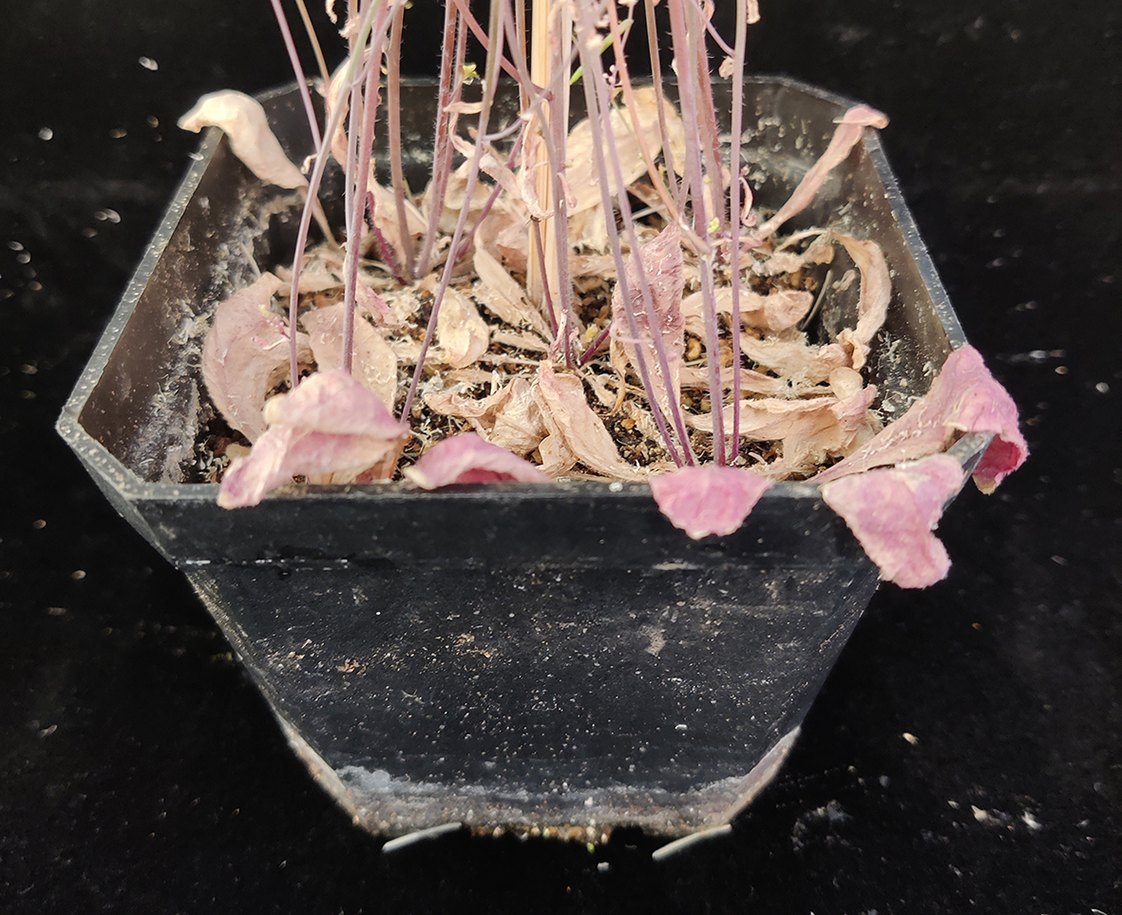

Supplement: Supplemental Information 2 [file peerj-11-16658-s002.zip › 7.27/Fig. 6/oe-3 (1).png]

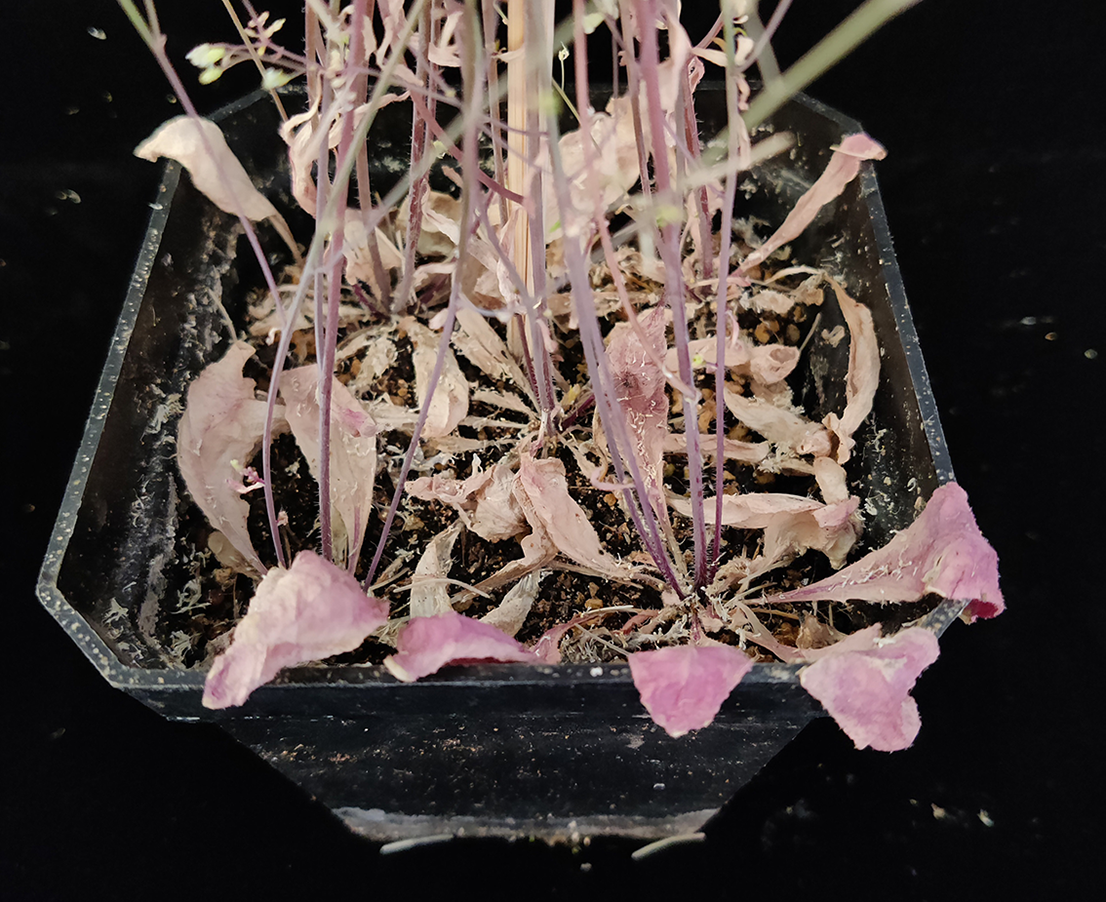

Supplement: Supplemental Information 2 [file peerj-11-16658-s002.zip › 7.27/Fig. 6/oe-4 (1).png]

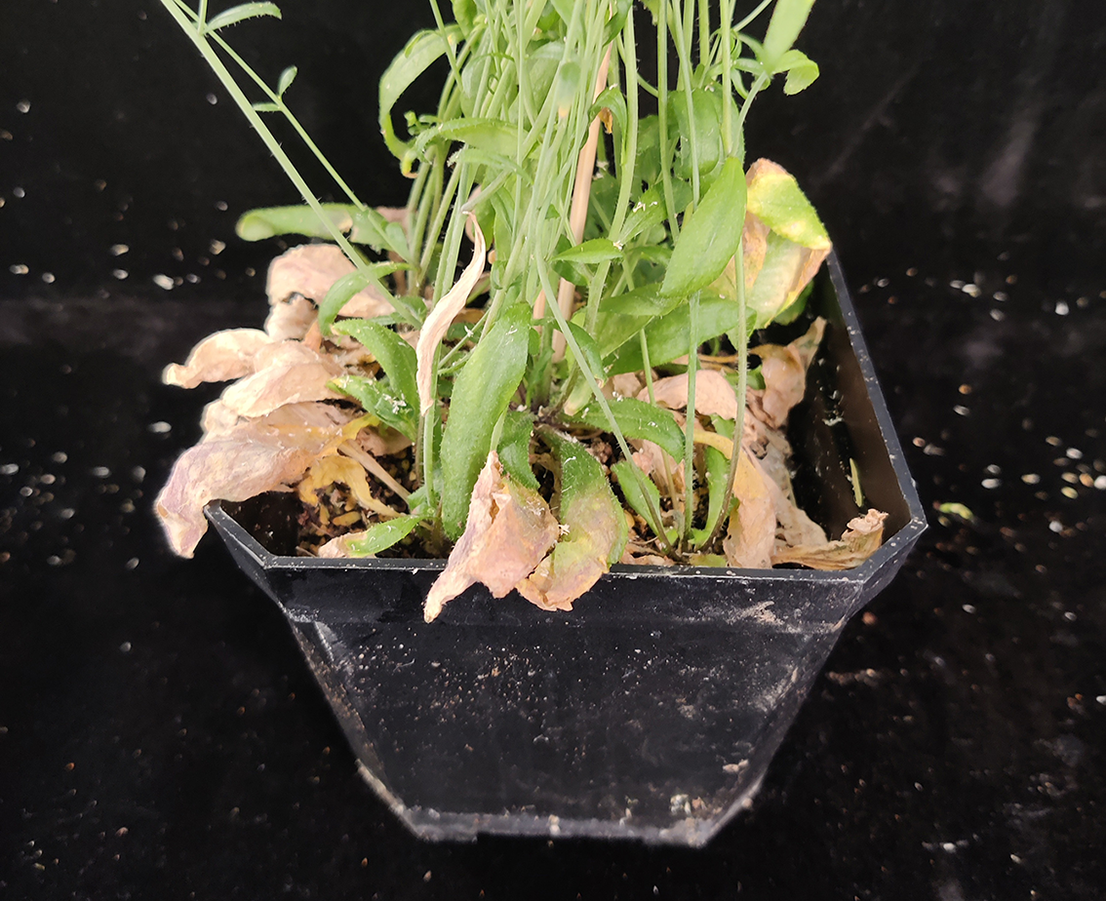

Supplement: Supplemental Information 2 [file peerj-11-16658-s002.zip › 7.27/Fig. 6/wt (1).png]
